# Transcriptomic profiling provides molecular insights into hydrogen peroxide-induced adventitious rooting in mung bean seedlings

**DOI:** 10.1186/s12864-017-3576-y

**Published:** 2017-02-17

**Authors:** Shi-Weng Li, Yan Leng, Rui-Fang Shi

**Affiliations:** School of Environmental and Municipal Engineering, Key Laboratory of Extreme Environmental Microbial Resources and Engineering, Gansu Province, Lanzhou Jiaotong University, 88 West Anning Road, Lanzhou, 730070 People’s Republic of China

**Keywords:** *Vigna radiata* (L.) R. Wilczek, Adventitious roots, Gene expression, Hydrogen peroxide (H_2_O_2_), Transcriptome, RNA-Seq

## Abstract

**Background:**

Hydrogen peroxide (H_2_O_2_) has been known to function as a signalling molecule involved in the modulation of various physiological processes in plants. H_2_O_2_ has been shown to act as a promoter during adventitious root formation in hypocotyl cuttings. In this study, RNA-Seq was performed to reveal the molecular mechanisms underlying H_2_O_2_-induced adventitious rooting.

**Results:**

RNA-Seq data revealed that H_2_O_2_ treatment greatly increased the numbers of clean reads and expressed genes and abundance of gene expression relative to the water treatment. Gene Ontology (GO) and Kyoto Encyclopedia of Genes and Genomes (KEGG) pathway enrichment analyses indicated that a profound change in gene function occurred in the 6-h H_2_O_2_ treatment and that H_2_O_2_ mainly enhanced gene expression levels at the 6-h time point but reduced gene expression levels at the 24-h time point compared with the water treatment. In total, 4579 differentially expressed (2-fold change > 2) unigenes (DEGs), of which 78.3% were up-regulated and 21.7% were down-regulated; 3525 DEGs, of which 64.0% were up-regulated and 36.0% were down-regulated; and 7383 DEGs, of which 40.8% were up-regulated and 59.2% were down-regulated were selected in the 6-h, 24-h, and from 6- to 24-h treatments, respectively. The number of DEGs in the 6-h treatment was 29.9% higher than that in the 24-h treatment. The functions of the most highly regulated genes were associated with stress response, cell redox homeostasis and oxidative stress response, cell wall loosening and modification, metabolic processes, and transcription factors (TFs), as well as plant hormone signalling, including auxin, ethylene, cytokinin, gibberellin, and abscisic acid pathways. Notably, a large number of genes encoding for heat shock proteins (HSPs) and heat shock transcription factors (HSFs) were significantly up-regulated during H_2_O_2_ treatments. Furthermore, real-time quantitative PCR (qRT-PCR) results showed that, during H_2_O_2_ treatments, the expression levels of *ARFs*, *IAAs*, *AUXs*, *NACs*, *RD22*, *AHKs*, *MYBs, PIN1*, *AUX15A*, *LBD29*, *LBD41*, *ADH1b,* and *QORL* were significantly up-regulated at the 6- and/or 24-h time points. In contrast, *PER1* and *PER2* were significantly down-regulated by H_2_O_2_ treatment. These qRT-PCR results strongly correlated with the RNA-Seq data.

**Conclusions:**

Using RNA-Seq and qRT-PCR techniques, we analysed the global changes in gene expression and functional profiling during H_2_O_2_-induced adventitious rooting in mung bean seedlings. These results strengthen the current understanding of H_2_O_2_-induced adventitious rooting and the molecular traits of H_2_O_2_ priming in plants.

**Electronic supplementary material:**

The online version of this article (doi:10.1186/s12864-017-3576-y) contains supplementary material, which is available to authorized users.

## Background

The plant root system is composed of primary roots, lateral roots, and adventitious roots. Adventitious roots refer to roots that form from any non-root tissue. These roots can generates from the tissues of old roots, stems or leaves during normal development to replenish and strengthen the roles of primary roots and in response to stresses [1]. The formation of adventitious roots is an important technique for commercial propagation of homogeneous horticultural plants and forest trees [2]. In addition, the process of adventitious root formation provides an ideal experimental system with which to study the important physiological and molecular events that occur during the tissue dedifferentiation and morphogenesis [3]. Many exogenous and endogenous factors that play important roles in adventitious rooting have been extensively studied using physiological and molecular methods. Auxin is known to play a critical role in inducing cell dedifferentiation and root primordia formation in cuttings. Other molecules, such as hydrogen peroxide (H_2_O_2_) and nitric oxide (NO) that produced by cells in response to various stresses, also act as signalling molecules that are involved in modulating of the induction and initiation of adventitious root formation [4]. H_2_O_2_ and NO are considered as key components of the molecular control of adventitious root formation in cuttings responding to wounding and auxin [5]. Li *et al.* reported that exogenous application of H_2_O_2_ promoted adventitious rooting in mung bean and cucumber hypocotyl cuttings and that H_2_O_2_ acts as a downstream signalling molecule in auxin-induced adventitious rooting [6, 7]. Yang demonstrated that H_2_O_2_ acts as a downstream modulator in SA-triggered adventitious root formation in mung bean seedlings [8]. However, the molecular mechanisms by which H_2_O_2_ promotes adventitious root development remain elusive.

H_2_O_2_ has been well known to act as a key signalling molecule that mediates a wide range of physiological processes [9], including senescence [10], photorespiration and photosynthesis [11], stomatal movement [12], cell cycle [13], seed germination [14], programmed cell death (PCD) [15], growth and development [16], as well as coordination of responses to biotic and abiotic stresses [17]. A number of studies have demonstrated that pre-treatment with an appropriate level of H_2_O_2_, now known as H_2_O_2_ priming, can enhance tolerance to abiotic and biotic stresses through the modulation of multiple physiological processes and stress-responsive pathways [17]. H_2_O_2_ pre-treatment induces increases in reactive oxygen species (ROS) scavenging enzyme activities and modulates the expression of genes involved in ROS control; signal transduction; transcriptional regulation; and protein, carbohydrate, and lipid metabolism [18]. For example, exogenously applied H_2_O_2_ increased the expression of genes encoding ROS scavenging proteins, such as genes *GPX* (glutathione peroxidase) and *GST* (glutathione S-transferase) [19]; *SOD* (superoxide dismutase) [20]; *GPX1* (glutathione peroxidase) [21]; *CAT1*, *CAT2* and *CAT3* (catalase) [20, 22]; *GST1* [23]; *APX* (ascorbate peroxidase) [14, 20]; *GR* (glutathione reductase) [20]; and *AOX1a* (alternative oxidase) [23], as well as genes encoding proteins required for peroxisome biogenesis [24]. Another study demonstrated that H_2_O_2_ induced the expression of genes encoding proteins related to plant signalling and development, cell elongation and division, and cell cycle control, including 14-3-3-like protein, profiling 4, proteasome-alpha-type-5, translationally controlled tumour protein homolog, and benzoquinone reductase, but reduced the expression of genes encoding lectin, albumin-2, ABA-responsive protein ABR18, and cell-autonomous heat shock cognate protein 70 [14]. H_2_O_2_ also increased the expression of genes encoding pyrroline-5-carboxylate synthase, sucrose- phosphate synthase, and small heat shock proteins [25]. Furthermore, exogenous H_2_O_2_ has been shown to be involved in the activation of mitogen-activated protein kinase (MAPK) cascade pathways [26]. H_2_O_2_ strongly activates MAPK4, MAPK12 [27], MPK1/2 [20], MPK6, and nucleotide diphosphate kinase 2 (NDPK2), which specifically interacts with MPK3 and MPK6, thereby regulating the cellular redox state [28]. However, little molecular information is available regarding H_2_O_2_-induced adventitious root development.

In our previous studies, we reported that exogenous H_2_O_2_ strongly promoted adventitious rooting in mung bean hypocotyl cuttings [6]. Recently, we characterized the mung bean transcriptome and gene expression profiles during the induction and initiation stages of adventitious rooting in water-treated [29] or IBA-treated [30] mung bean hypocotyl cuttings using RNA-Seq technology. In the present study, RNA-Seq technology and qRT-PCR were exploited to reveal the transcriptome of H_2_O_2_ promotion adventitious rooting in mung bean hypocotyl cuttings. Our main objective of the present study was to analyse the gene profiles and metabolic pathways that specifically responded to exogenous H_2_O_2_ treatment and further to uncover the molecular basis of H_2_O_2_ priming and promotion of adventitious rooting in plants.

## Results and discussion

### RNA sequencing, reads mapping, de novo assembly, unigene determination, and gene expression abundance analysis

**Fig. 1:**
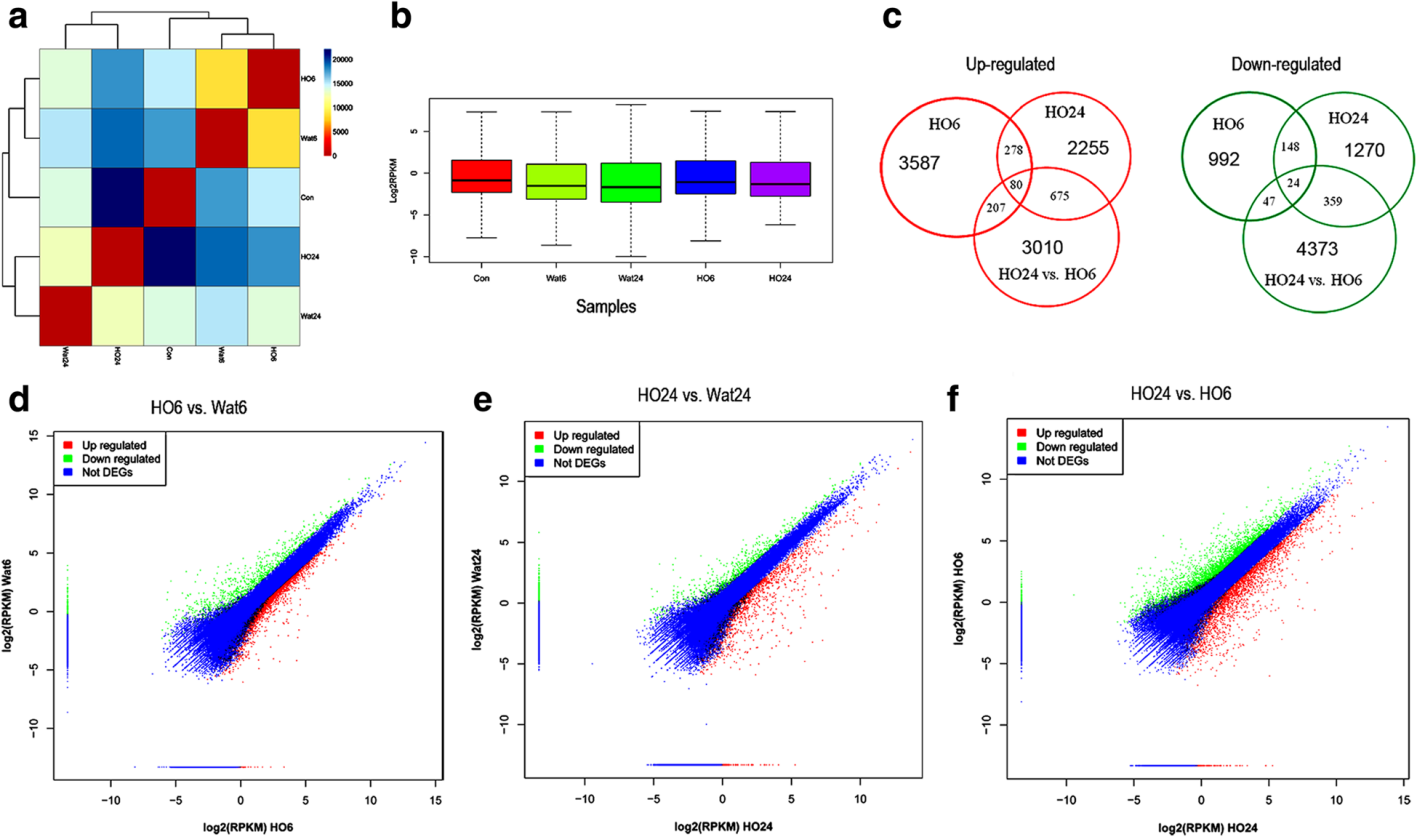
Analysis of RNA-Seq data and comparison of expressed unigenes between the samples. **a** Heatmap of the samples based on RPKM data. **b** Boxplot of the samples based on RPKM data. **c** Venn maps of up-regulated and down-regulated genes between the sample pairs. **d-f** Scatter plot comparing the gene expression levels in HO6 vs. Wat6, HO24 vs. Wat24, and HO24 vs. HO6

In this experiment, the hypocotyl tissues that were treated with 10 mM H_2_O_2_ for 6 h or 24 h, designated as HO6 and HO24, which represent the induction stage and initiation stage of adventitious root development in mung bean seedlings, respectively [29, 30], were separately sampled for total RNA extraction, cDNA library construction, and Illumina Solexa RNA paired-end sequencing. The sequencing data and mapping results are listed in Table 1. This sequencing generated approximately 93% of the high-quality reads from the samples, and approximately 93% of the quality reads mapped uniquely to the reference sequences that were constructed in our previous study [29, 30]. Using the Chrysalis cluster module of TRINITY, we identified a total of 78,697 unigenes from all the samples [29, 30]. The sequencing data were used for bioinformatics analysis with the data from the same batch samples treated with water for 6 h (Wat6) or 24 h (Wat24) that were reported in our previous study [29]. Data analysis showed that the number of clean reads and expressed genes were increased by 33.47% in HO6 and 2.98% in HO24 and by 9.21% (6142 genes) in HO6 and 7.12% (4602 genes) in HO24 compared with Wat6 and Wat24, respectively [29]; and the expressed gene number were increased by 4.71% and 2.0% compared with 6- and 24-h IBA treatments, respectively [30]. This result indicates that H_2_O_2_ treatment greatly increased the number of expressed genes during two time points in comparison to the water and IBA treatments, particularly in the sample treated for 6 h. Cluster analysis of the samples showed that the HO24 and HO6 treatments separately grouped with Wat24 and Wat6, suggesting that the changes in gene expression abundance mainly depended on the time duration rather than the treatments during adventitious rooting in mung bean seedlings (Fig. 1a). Furthermore, gene expression abundance was increased in HO6 and HO24 relative to Wat6 and Wat24 (Fig. 1b).

**Table 1:** Data obtained from RNA-Seq

Samples	Raw sequences	Mean length	Clean reads	Clean reads ratio (%)	Mean length	Mapped reads	Mapped ratio (%)	Expressed genes	Total genes	Expressed ratio (%)
HO6	80,070,690	100	74,186,441	92.65	95.16	69,199,238	93.28	72,805	78,697	92.51%
HO24	61,763,482	100	57,227,917	92.66	95.09	53,210,685	92.98	69,282	78,697	88.04%

**Fig. 2:**
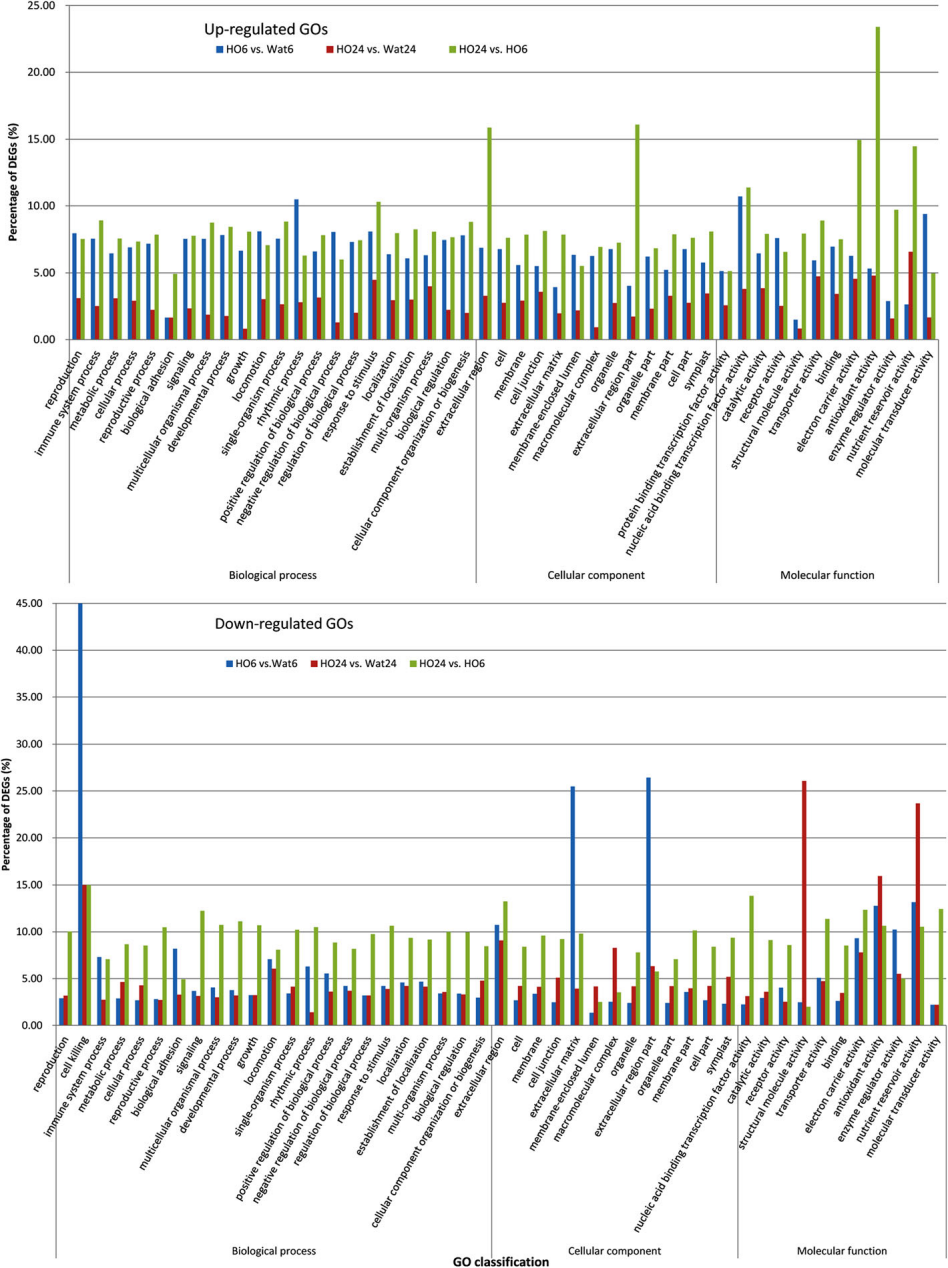
The distribution of GO terms enriched in the sample pairs

### Functional enrichment analysis of the genes expressed during H_2_O_2_-induced adventitious rooting

The functional enrichment results of the unigenes from the Clusters of Orthologous Groups for Eukaryotic Complete Genomes (KOG) database were clustered using Blast2GO software [31] and WEGO software [32] and designated as GO terms. The differentially expressed unigenes with 2-fold changes (log2 > 1) between the samples under each GO category were further filtered. The statistical analysis results are listed in Additional file 1 and Table 2. Table 2 shows that the total number of GOs enriched in HO6 is 39.6% higher than that in HO24 and that there are 78.5% and 10.9% more up- and down-regulated GOs in HO6 than in HO24, respectively, indicating that a profound change in gene function occurred during the 6-h H_2_O_2_ treatment (Table 2). There are 21.7% more up-regulated GOs than down-regulated GOs in HO6 and 32.3% more down-regulated GOs than up-regulated GOs in HO24, suggesting that H_2_O_2_ mainly enhanced gene expression levels at the 6-h time point but reduced gene levels at the 24-h time point.

**Table 2:** Changes in the GO categories and KO pathways in the sample groups

Samples		Enriched differential GOs	Enriched differential KOs
HO6 vs. Wat6	up	3865	204
	down	3177	129
HO24 vs. Wat24	up	2165	168
	down	2865	155
HO24 vs. HO6	up	4083	248
	down	3744	234

We further analysed the ratio of differentially expressed genes (DEGs) in each up- and down-regulated GO category (Fig. 2). In the up-regulated GO group, nearly all of the DEG ratios in HO6 were greater than those in HO24, while the opposite result was observed in the down-regulated GO group, suggesting that most of the DEGs were significantly up-regulated by the 6-h H_2_O_2_ treatment. The DEG ratios under the GO terms extracellular region and extracellular region part in cellular component, as well as electron carrier activity, antioxidant activity, enzyme regulator activity, and nutrient reservoir activity in molecular function were strongly increased from HO6 to HO24 in the up-regulated GO group, suggesting the DEGs in these GO terms were significantly up-regulated from 6 h to 24 h of treatment. In the down-regulated GO group, DEG ratios under the GO terms cell killing in biological process and extracellular region part in cellular component were significantly increased in HO6, while DEG ratios under the GO terms antioxidant activity, structural molecule activity, and nutrient reservoir activity in molecular function were significantly increased in HO24, suggesting that the DEGs in these GO terms were down-regulated by H_2_O_2_ treatment for 6 h and 24 h, respectively.

The most significantly up- and down-regulated GOs that specifically responded to H_2_O_2_ treatment are listed in Additional file 1 and Table 3. In Summary, H_2_O_2_ treatment for 6 h significantly up-regulated the GOs associated with the functions cellular component movement, transcription, DNA synthesis, cell cycle, hydrolase activity, and response to stress and down-regulated the GOs associated with oxidoreductase, cellular respiration and oxidative phosphorylation, and lipid transport. H_2_O_2_ treatment for 24 h significantly up-regulated the GOs associated with oxidoreductase activity, transcription, isoflavonoid biosynthetic process, and ethylene biosynthetic process and down-regulated the GOs related to protein synthesis, cellular biosynthetic process, and cell wall organization. Interestingly, 6- and 24-h IBA treatment mainly up-regulated the GOs associated with auxin signalling, ribosome assembly and protein synthesis, and nucleolus [30]; whereas, H_2_O_2_ mainly up-regulated cellular component movement, transcription, and response to stress. With respect to the down-regulated GOs, the same were detected in both IBA and H_2_O_2_ treated samples, such as extracellular region, oxidoreductase activity, ribosome, except photosynthesis. Furthermore, cell wall loosening was up-regulated while cell wall organization was down-regulated in both IBA and H_2_O_2_ treated samples.

**Table 3:** Summary of the top differentially regulated GOs in the sample groups

Samples	Up-regulated GOs	Down-regulated GOs
HO6 vs. Wat6	(1) cellular component movement, microtubule, cytoskeleton (2) transcription, DNA binding, sequence-specific DNA binding, RNA biosynthetic process and regulation (3) cell cycle, mitosis, organelle organization and localization (4) hydrolase activity (5) response to stress (6) oligosaccharide metabolic, nitrogen compound biosynthetic process, and fatty acid ligase activity	(1) extracellular region (2) oxidoreductase, peroxidase, and antioxidant activity, response to wounding and oxidative stress (3) endopeptidase inhibitor and enzyme inhibitor activity (4) electron transport chain, cellular respiration and oxidative phosphorylation (5) negative regulation of hydrolase activity (6) lipid transport and localization
HO24 vs. Wat24	(1) oxidoreductase activity, response to stress, response to oxidative stress, glutathione transferase activity (2) coenzyme binding (3) acyl groups transferase activity (4) DNA integration, DNA-directed DNA polymerase activity (5) RNA-directed DNA polymerase activity (6) aspartic-type endopeptidase activity (7) isoflavonoid biosynthetic process (8) active transmembrane transporter activity (9) amino acid, oxylipin, raffinose, oxoacid, organic acid, alkene biosynthetic process (10) ethylene biosynthetic process	(1) ribosome, ribonucleoprotein complex (2) translation, translation elongation factor activity (3) extracellular region (4) cellular response to oxidative stress, peroxidase activity, oxidoreductase activity, antioxidant activity (5) cellular biosynthetic process (6) cell wall organization or biogenesis (7) lipid catabolic process (8) organelle assembly, mitotic spindle elongation (9) enzyme inhibitor activity, peptidase inhibitor activity (10) electron carrier activity
HO24 vs. HO6	(1) extracellular region (2) oxidoreductase activity, antioxidant activity, peroxidase activity, glutathione transferase activity (3) response to stress, response to oxidative stress, cellular response to reactive oxygen species (4) hydrogen peroxide catabolic and metabolic process, reactive oxygen species metabolic process (5) nucleosome, cell division, chromatin, mitosis, nuclear division (6) DNA packaging, DNA conformation change, chromatin assembly, protein-DNA complex assembly, chromatin assembly or disassembly, nucleosome assembly (7) response to inorganic and organic substance, organonitrogen compound, nitrogen compound, water deprivation, and wounding (8) cellular response to ethylene stimulus, ethylene mediated signalling pathway (9) cell wall (10) electron carrier activity	(1) secondary cell wall biogenesis, UDP-glucosyltransferase activity, cellulose synthase activity; carbohydrate, polysaccharide, lignin, xylan, beta-glucan, and polysaccharide biosynthetic process (2) response to auxin stimulus, auxin mediated signalling pathway (3) sequence-specific DNA binding transcription factor activity, transcription, RNA biosynthetic process, chromatin binding (4) lipid metabolic process, lipid biosynthetic process (5) response to organic substance (6) oxidoreductase activity (7) cell periphery (8) extracellular region (9) signal transduction (10) phenylpropanoid metabolic process

### KEGG pathway enrichment analysis

The KEGG pathways that were significantly regulated by H_2_O_2_ were enriched using the KEGG Automatic Annotation Server (KAAS) [33]. The results showed that there were 58.1% more up-regulated KOs (204) than down-regulated KOs (129) in HO6, suggesting that H_2_O_2_ treatment for 6 h mainly up-regulated certain metabolic pathways (Table 2). When compared with the water treatment, pathways such as glutathione metabolism, biosynthesis of amino acids, protein processing, DNA replication, purine metabolism, cutin, suberine and wax biosynthesis, and starch and sucrose metabolism were up-regulated, while pathways including oxidative phosphorylation, phenylalanine metabolism, photosynthesis, flavonoid biosynthesis, ribosome, plant hormone signal transduction, and phenylpropanoid biosynthesis were down-regulated by H_2_O_2_ treatment for 6 h or 24 h (Table 4). The enriched DEGs under the glutathione metabolism pathway are mainly genes coding for glutathione S-transferase, which is involved in cell redox homeostasis and plays a vital role in the responses to various stresses [34]. Phenylpropanoids and flavonoids are major plant secondary metabolites that have a wide variety of functions as structural, signalling, and stress response molecules [35]. The derivatives from the phenylpropanoid pathway are also involved in the regulation of cell division and differentiation [36] and stimulate in vitro rooting [37]. Limonene and pinene are involved in terpenoid biosynthesis. These results indicate that H_2_O_2_ significantly up-regulated the processes related to stress response, biosynthesis of amino acids, protein processing, and cell cycle but down-regulated the processes related to energy metabolism, photosynthesis, protein synthesis, lipid and carbohydrate metabolism, plant hormone signal transduction, and environmental adaptation.

**Table 4:** List of the top differentially regulated KOs in the sample groups

Treatments	KO ID	All genes	DEGs	*p*-value	FDR	Description
HO6 vs. Wat6 up-regulated	ko04744	11	4	0.002	0.176	purine metabolism
	ko00073	12	4	0.003	0.176	cutin, suberine and wax biosynthesis
	ko03030	47	8	0.004	0.176	DNA replication
	ko04141	145	16	0.007	0.247	protein processing in endoplasmic reticulum
	ko00500	119	13	0.016	0.402	starch and sucrose metabolism
HO6 vs. Wat6 down-regulated	ko00190	143	21	0.000	0.000	oxidative phosphorylation
	ko00360	57	8	0.001	0.008	phenylalanine metabolism
	ko00195	53	7	0.002	0.021	photosynthesis
	ko00941	17	4	0.002	0.023	flavonoid biosynthesis
	ko04075	130	10	0.012	0.096	plant hormone signal transduction
HO24 vs. Wat24 up-regulated	ko00480	39	9	0.000	0.000	glutathione metabolism
	ko01230	29	7	0.000	0.002	biosynthesis of amino acids
	ko04141	145	13	0.001	0.026	protein processing in endoplasmic reticulum
	ko00910	31	5	0.003	0.066	nitrogen metabolism
HO24 vs. Wat24 down-regulated	ko03010	337	116	0.000	0.000	ribosome
	ko00940	69	9	0.006	0.290	phenylpropanoid biosynthesis
	ko00908	11	3	0.014	0.535	zeatin biosynthesis
	ko00073	12	3	0.018	0.551	cutin, suberine and wax biosynthesis
HO24 vs. HO6 up-regulated	ko00940	69	21	0.000	0.000	phenylpropanoid biosynthesis
	ko04110	94	22	0.000	0.000	cell cycle
	ko00360	57	16	0.000	0.000	phenylalanine metabolism
	ko04626	77	15	0.000	0.008	plant-pathogen interaction
	ko00480	63	13	0.001	0.009	glutathione metabolism
	ko00903	25	7	0.002	0.021	limonene and pinene degradation
HO24 vs. HO6 down-regulated	ko00904	8	6	0.000	0.001	diterpenoid biosynthesis
	ko00500	119	21	0.000	0.009	starch and sucrose metabolism
	ko04712	23	8	0.000	0.009	circadian rhythm - plant
	ko04075	130	21	0.000	0.014	plant hormone signal transduction
	ko00592	26	8	0.000	0.014	α-linolenic acid metabolism
	ko00909	7	4	0.001	0.029	sesquiterpenoid and triterpenoid biosynthesis

### Global changes in differentially expressed genes (DEGs) at two time points during H_2_O_2_-induced adventitious rooting

**Fig. 3:**
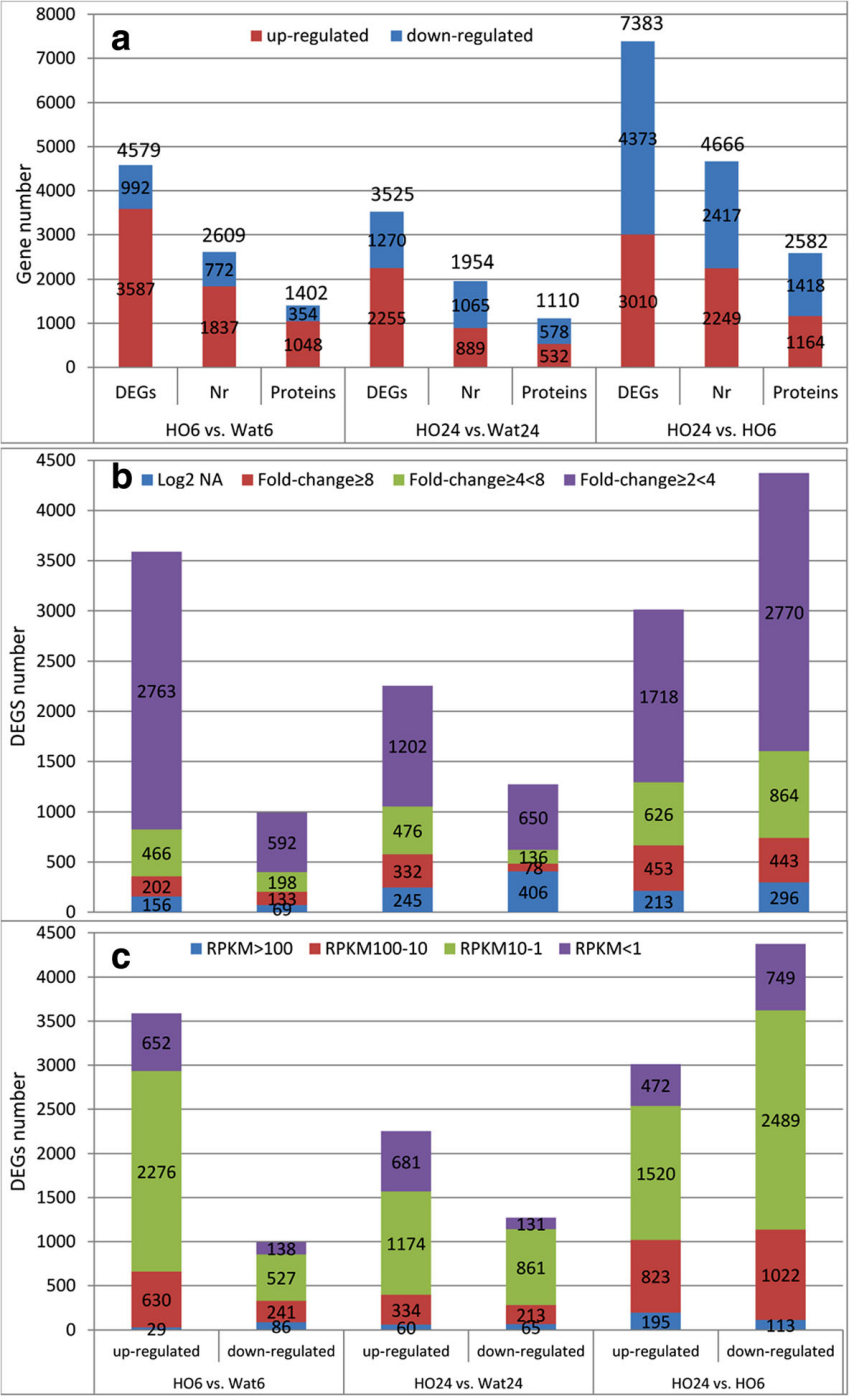
The distribution of DEGs in the sample pairs. **a**. Numbers of up- and down-regulated DEGs, Nr annotation, and putative proteins. **b**. Changes in DEG expression levels between the sample pairs. **c**. Distribution of DEG expression abundance between the sample pairs

To examine the gene expression profile specifically in response to H_2_O_2_ treatment, the gene expression levels were measured and analysed with a threshold of log2 ≥ 1 between the samples using the method described by the DEGseq R package [38]. In total, 4579 unigenes were identified, with 3587 (78.3%) up-regulated and 992 (21.7%) down-regulated; and 3525 unigenes, with 2255 (64.0%) up-regulated and 1270 (36.0%) down-regulated in HO6 vs. Wat6 and HO24 vs. Wat24, respectively (Figs. 1c and 3a). Of these DEGs, approximately 58% of the unigenes could be annotated using the Nr database, and 32% unigenes could be defined as a protein. The number of DEGs in HO6 vs. Wat6 was 29.9% higher than that in HO24 vs. Wat24, indicating that a great change in gene expression in response to H_2_O_2_ occurred at the 6-h time point. Additionally, the down-regulated DEGs in HO24 increased 3.4-fold more than that in HO6, indicating that H_2_O_2_ strongly down-regulated gene expression from 6 h to 24 h treatment (Fig. 1d, e, and f; Fig. 3a and b). The DEGs with 2- to 4-fold changes accounted for 73.3% and 52.5% in HO6 vs. Wat6 and HO24 vs. Wat24, respectively, and the numbers of specially regulated DEGs, DEGs with fold change ≥ 8, and DEGs with fold change ≥ 4 to <8 in HO24 vs. Wat24 was remarkably higher than those in HO6 vs. Wat6 (Fig. 3b). This result indicates that the 24-h treatment strongly enhanced the expression levels of certain genes. Furthermore, the abundance of DEGs with RPKM > 100, RPKM = 10-100, and RPKM = 1-10 accounted for 2.5%, 19.0%, and 61.2% in HO6 vs. Wat6 and 3.5%, 15.5%, and 57.7% in HO24 vs. Wat24, respectively. This analysis shows that the time course of H_2_O_2_ treatments had no significant effect on the DEG abundance (Fig. 3c).

### Gene expression patterns during H_2_O_2_-induced adventitious rooting

**Fig. 4:**
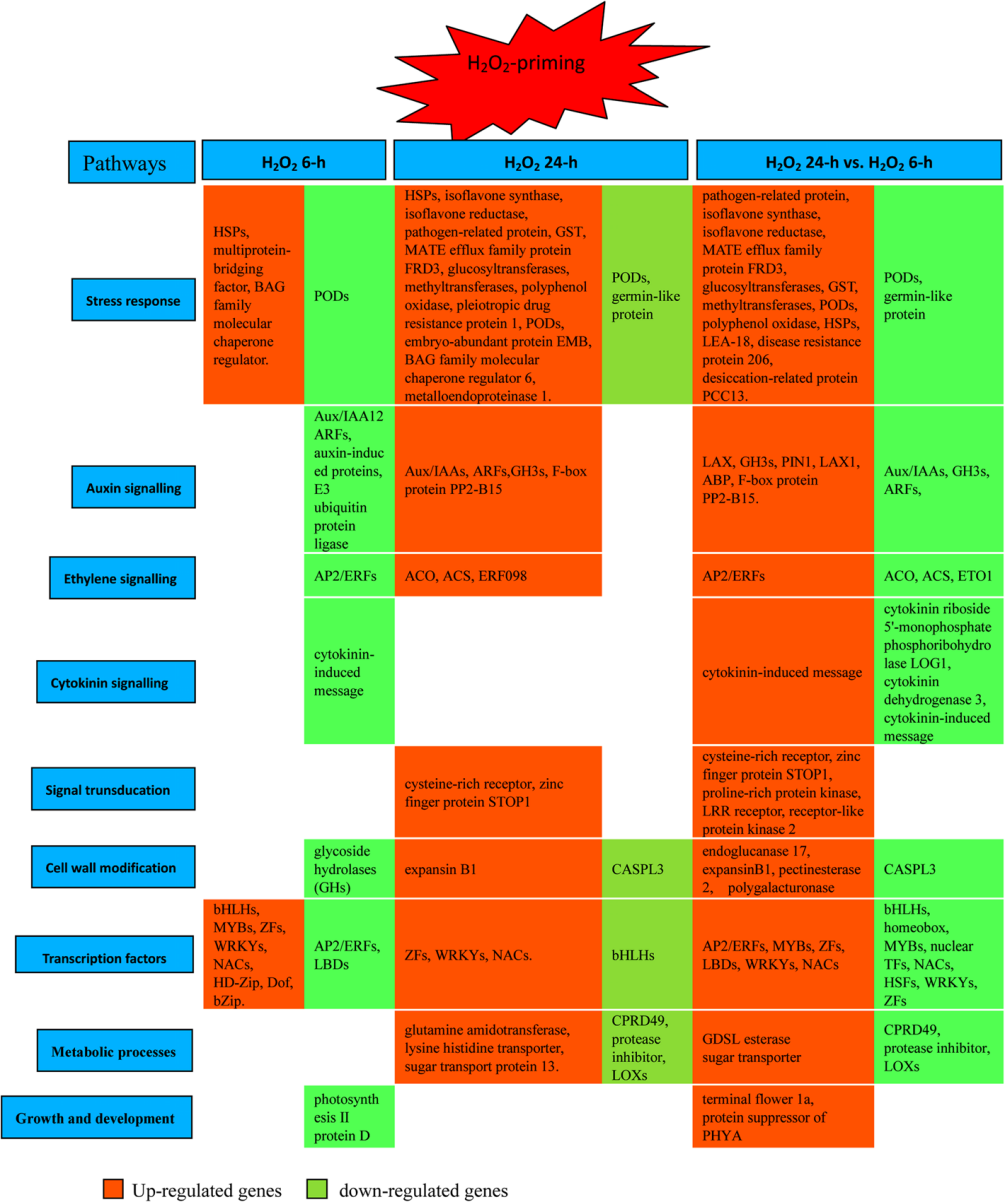
Overview of the most highly regulated genes in response to H_2_O_2_-priming during H_2_O_2_-induced adventitious rooting

To gain insight into the DEGs patterns that specifically responded to H_2_O_2_ treatment during two stages of adventitious rooting, the unigenes defined as unknown, hypothetical protein, uncharacterized protein, predicted protein, and unnamed protein in the database were filtered out; thus only the genes that had been assigned to proteins in the database remained. The remaining DEGs were further filtered, retaining those with RPKM ≥ 10 and fold change ≥ 2. The results are listed in Additional files 2, 3, 4, 5, 6, and 7. There were 530 DEGs in total, of which 364 were up-regulated 2- to 55-fold and 166 were down-regulated 2- to 13-fold in HO6 vs. Wat6; 396 DEGs, of which 235 were up-regulated 2- to 1789-fold and 161 were down-regulated 2- to 134-fold in HO24 vs. Wat24. To further understand the functions of the DEGs with RPKM > 10 and fold change > 8, each DEG was searched in the UniProt Knowledgebase (http://www.uniprot.org) [35]. The search result was summarised in Fig. 4, indicating that these DEGs can be clustered into functional categories as stress response, cell redox homeostasis and oxidative stress response, plant hormone (auxin, ethylene, and cytokinin) signalling, signal transduction, cell wall modification, transcription factors, metabolic processes, and growth and development. Here, we focused on the DEGs with a RPKM ≥ 10 and a fold change ≥8 in the samples.

### Top significantly regulated DEGs during root induction stage with H_2_O_2_ treatment

Sixteen DEGs were up-regulated 8- to 55-fold in HO6 vs. Wat6, including 11 small heat shock protein (HSP)-like genes, a BAG family molecular chaperone regulator 6-like, a germin-like protein, a multiprotein-bridging factor 1c-like, and an extensin class 1 protein (Additional file 2). These proteins are known to be involved in the response to various stresses, including H_2_O_2_ [35]. Small heat shock protein and chaperone regulator, which is a modulator of chaperone activity, are involved in protein assembly and folding in response to stress [35]. Multiprotein-bridging factor is a transcriptional coactivator that functions in the tolerance to heat and osmotic stress by partially activating the ethylene-response signal transduction pathway [35]. Extensin serves as structural constituent of the cell wall and is expressed in response to wounding [39]. A total of 10 DEGs were down-regulated 8- to 13-fold in HO6 vs. Wat6, including two peroxidase-like isoform genes, a cysteine-rich repeat secretory protein, a yieldin precursor, an auxin-responsive protein IAA12-like, a photosystem II protein D1, a 7-ethoxycoumarin O-deethylase-like (flavonoid 3′-monooxygenase), an E3 ubiquitin-protein ligase PUB23-like, and a polygalacturonase PG1 precursor gene (Additional file 3). The genes coding for cysteine-rich repeat secretory protein, peroxidase, and E3 ubiquitin-protein ligase are all involved in the stress response [35]. The isoform of peroxidase is also involved in the synthesis and degradation of lignin, suberization, and auxin catabolism [35]. Yieldin and polygalacturonase PG1 are glycoside hydrolases (GH) that are involved in cell wall loosening [35]. Photosystem II protein D1 functions in pathway of photosynthetic electron transport in photosystem II [35]. The auxin-responsive protein IAA12, a member of AUX/IAA family, acts as a repressor of auxin signalling and plays an important role during the different phases of adventitious root formation [5]. These results indicate that, during induction of adventitious rooting, H_2_O_2_ treatment strongly induced the expression of genes related to stress response, including protein processing and cell wall remodeling in response to stress; whereas, down-regulated genes related to oxidative stress, cell wall loosening, and photosynthesis. The down-regulation of oxidative stress-related genes suggests that H_2_O_2_ might alleviate the oxidative stress occurred in the process. Moreover, H_2_O_2_ initiated auxin signalling leading to rooting via down-regulating the *AUX/IAA* gene at this stage.

### Top significantly regulated DEGs during root initiation stage with 24-h H_2_O_2_ treatment

A total of 58 DEGs were up-regulated 8- to 1789-fold in HO24 vs. Wat24, including seven small heat shock protein (HSP)-like genes, three auxin-induced protein genes, three isoflavone synthase genes, three pathogen-related protein genes, two glutamine amidotransferase-like protein RP404-like genes, two galactose oxidase-like genes, two glutathione S-transferase (GST) genes, two isoflavone reductase-like genes, two mannitol dehydrogenase-like genes, two lysine histidine transporter 1-like genes, a multidrug and toxic compound extrusion (MATE) efflux family protein FRD3-like, a pterocarpan reductase, a F-box protein PP2-B15-like, a formate dehydrogenase, a limonoid UDP-glucosyltransferase-like, a hydroquinone glucosyltransferase-like isoform 1, an anthranilate N-methyltransferase-like, a cysteine-rich receptor-like protein kinase 29-like (CRK29), an ERF098-like, a germin-like protein, a quinate hydroxycinnamoyltransxferase-like, an expansin-like B1-like, a zinc finger protein STOP1 homolog, a N-hydroxycinnamoyl/benzoyltransferase, a chitinase class 1, a UDP-glycosyltransferase 73B3-like, an isoliquiritigenin 2′-O-methyltransferase-like, a polyphenol oxidase, a pleiotropic drug resistance protein 1-like, a cationic peroxidase 1-like, a sugar transport protein 13-like, a basic 7S globulin-like, an embryo-abundant protein EMB, a BAG family molecular chaperone regulator 6-like, a metalloendoproteinase 1-like, a syntaxin-24-like, a glutamate decarboxylase 1-like, a theobromine synthase 2-like, a nitronate monooxygenase-like, and a nonsymbiotic haemoglobin gene (Additional file 4). Of these genes, HSPs, pathogen-related protein, MATE efflux family protein, pleiotropic drug resistance protein, BAG family molecular chaperone regulator, CRK29, germin-like protein, chitinase class 1, N-hydroxycinnamoyl/benzoyltransferase, a metalloendoproteinase 1-like, zinc finger protein STOP1, and nitronate monooxygenase are all involved in stress and defense response [35]. N-hydroxycinnamoyl/benzoyltransferase catalyzes the formation of phytoalexin, which are essential for the expression of disease resistance [35]. Metalloendoproteinase may play a role in the degradation and remodeling of the extracellular matrix during development or in response to stresses [35]. Zinc finger protein STOP1 acts as a transcription factor that was showed to be involved in aluminum tolerance [35]. Nitronate monooxygenase acts as a ferredoxin-dependent glutamate synthases, which have been implicated in a number of functions [35]. GST, isoflavone synthase, galactose oxidase, isoflavone reductase, pterocarpan reductase, mannitol dehydrogenase, formate dehydrogenase, polyphenol oxidase, and cationic peroxidase play vital roles in the responses to various stresses and cell redox homeostasis and were highly induced by exogenously applied H_2_O_2_ [19] and IBA during IBA-induced adventitious rooting [30]. GSTs are widely studied versatile proteins that are involved in hydroxyperoxide detoxification [40], cellular redox homeostasis [40], and stress signalling proteins [41]. GSTs also function as non-catalytic auxin carriers, thus contributing to hormone homeostasis [40]. Isoflavone synthase belongs to the cytochrome P450 family enzymes, which are important for the biosynthesis of several compounds such as hormones, defensive compounds and fatty acids [35]. Pterocarpan reductase (also isoflavone reductase), isoflavone reductase homologue A622, and isoflavone reductase are involved in the pathway of pterocarpan phytoalexin biosynthesis and cell redox homeostasis [35]. The auxin-induced protein, an Aux/IAA protein family member, and F-box protein PP2-B15 are involved in auxin signaling pathways [35]. Glutamine amidotransferase catalyses purine biosynthesis from glutamine and is required for chloroplast biogenesis and cell division [35]. Limonoid UDP-glucosyltransferase, hydroquinone glucosyltransferase, and UDP-glycosyltransferase 73B3 belong to a large family of membrane-bound microsomal enzymes which catalyse the transfer of glucuronic acid to a wide variety of exogenous and endogenous lipophilic substrates. These enzymes are of major importance in the detoxification and subsequent elimination of xenobiotics such as drugs and carcinogens [35]. Anthranilate N-methyltransferase, isoliquiritigenin 2′-O-methyltransferase, embryo-abundant protein EMB, and theobromine synthase are methyltransferase family enzymes, mostly S-adenosylmethionine (SAM)-dependent methyltransferase, which utilise the ubiquitous methyl donor SAM as a cofactor to methylate small molecules, lipids, proteins, and nucleic acids, and therefore involved in many essential cellular processes including biosynthesis, signal transduction, protein repair, chromatin regulation and gene silencing [35]. ERF098 may be involved in the regulation of gene expression by stress factors and by components of stress signal transduction pathways [35]. Quinate hydroxycinnamoyltransferase is an acyltransferase involved in the biosynthesis of lignin [35]. The other proteins such as sugar transport protein 13, basic 7S globulin (an aspartic-type endopeptidase), lysine histidine transporter 1, syntaxin-24 (a vesicle trafficking protein), glutamate decarboxylase 1, and nonsymbiotic haemoglobin (an electron transfer) are involved in nutrients and energy metabolic processes. Interestingly, the gene coding for MATE efflux family protein FRD3-like was up-regulated 1789-fold after 24 h of treatment. This protein belongs to a secondary transporter family [42, 43], which plays important roles in a wide range of biological processes in plants [44] and which has been shown to mediate the citrate efflux and iron transport to confer plant tolerance to Al toxicity [45]. Seven genes coding for small heat shock protein-like genes and a BAG family molecular chaperone regulator 6-like protein gene were highly up-regulated after 24 h of treatment. These proteins may act as protein chaperones that can protect other proteins against heat-induced denaturation and aggregation and are involved in plant defence processes [35]. Briefly, the above results indicate that, during initiation stage of adventitious rooting, H_2_O_2_ treatment greatly up-regulated the expression of genes coding for proteins that function in various stress responses, thus improving cell tolerance to stress for further development.

### Top significantly regulated DEGs from root induction stage to initiation stage with H_2_O_2_ treatment

A total of 68 genes were up-regulated 8- to 2193-fold in HO24 vs. HO6. Among those genes, 34 genes were up-regulated in both HO24 vs. Wat 24 and HO24 vs. HO6, including three AP2-ERF086-like genes, three mannitol dehydrogenase-like genes, two pathogen-related protein genes, two isoflavone synthase 2 genes, two galactose oxidase-like genes, a MATE efflux family protein FRD3-like, a cysteine-rich receptor-like protein kinase 29-like, a germin-like protein subfamily 1 member 7-like, a limonoid UDP-glucosyltransferase-like, a metalloendoproteinase 1-like, a syntaxin-24-like, a glutathione S-transferase, an anthranilate N-methyltransferase-like, a cationic peroxidase 1-like, a polyphenol oxidase A1, a chitinase, a N-hydroxycinnamoyl/benzoyltransferase, a hydroxycinnamoyl-coenzyme A shikimate/quinate hydroxycinnamoyltransferase-like, a bidirectional sugar transporter SWEET3-like, a small heat shock proteins, a LEA-18, an acidic endochitinase, an isoflavone reductase, a F-box protein PP2-B15, a pterocarpan reductase, a zinc finger protein STOP1 homolog, and disease resistance response protein 206-like genes. Clearly, these genes are associated with stress and defense response, ethylene pathway, cell redox homeostasis, and protein processing during the process. The other 34 genes were up-regulated only in HO24 vs. HO6, including two vignain-like genes, two reticuline oxidase-like protein-like genes, two proline-rich protein kinase genes, two LRR receptor-like serine/threonine-protein kinase genes, a GDSL esterase/lipase 5-like, a endoglucanase 17-like, an expansin-like B1-like, a pectinesterase 2-like, a homogentisate phytyltransferase 1, a desiccation-related protein PCC13-62-like, a peroxidase C3-like isoform 3, a laccase-7-like, a 4,5-DOPA dioxygenase extradiol-like protein-like, a patatin group A-3-like, a polygalacturonase PG1, an annexin-like protein RJ4, a receptor-like protein kinase 2-like, an auxin transporter-like protein 1-like, a terminal flower 1a, a gibberellic acid-stimulated protein 1, a ferritin-3, auxin-responsive GH3 product, a 21 kDa protein-like, a beta-fructofuranosidase, cell wall isozyme-like isoform 1, a 7-ethoxycoumarin O-deethylase-like, a polygalacturonase At1g48100 isoform 1, a protein suppressor of PHYA-105 1-like, a galactinol--sucrose galactosyltransferase-like, a mannan endo-1,4-beta-mannosidase 4-like, and a blue copper protein-like gene (Additional file 6). Of these genes, endoglucanase 17, pectinesterase 2, laccase-7, polygalacturonase PG1, beta-fructofuranosidase, polygalacturonase At1g48100 isoform 1, cell wall isozyme-like isoform 1, a mannan endo-1,4-beta-mannosidase 4, galactinol-sucrose galactosyltransferase (a glycoside hydrolase) are involved in cell wall loosening [35]. Proline-rich protein kinase, LRR receptor-like serine/threonine-protein kinase, and receptor-like protein kinase 2 are involved in stress signalling [35]. Reticuline oxidase, homogentisate phytyltransferase 1, desiccation-related protein PCC13-62, peroxidase C3, 4,5-DOPA dioxygenase extradiol-like protein, annexin-like protein RJ4, and 7-ethoxycoumarin O-deethylase (flavonoid 3′-monooxygenase, a cytochrome P450 family member) are involved in flavonoid biosynthesis and in stress response [35]. Vignain (a cysteine-type peptidase), patatin group A-3 (a phospholipase), and GDSL esterase/lipase 5 are involved in protein and lipid degradation and plant defences [35]. Annexin-like protein RJ4 is an annexins family protein that binds to phospholipids in a calcium-dependent manner and responds to reactive oxygen species [35]. The auxin transporter-like protein 1 acts as a carrier protein that is involved in proton-driven auxin influx and auxin gradient formation [35]. The auxin-responsive GH3 product belongs to IAA-amido synthetases that negatively regulate the levels of free IAA by conjugating IAA to amino acids [46]. The terminal flower 1a protein was shown to prevents the expression of ‘APETALA1’ and ‘LEAFY’ which are transcription factors that promotes early floral meristem identity is involved in cell differentiation [35]. The protein suppressor of PHYA-105 1-like was involved in suppression of photomorphogenesis in dark-grown seedlings and is required for normal elongation growth of adult plants [35]. The gibberellic acid-stimulated protein 1 has some role in plant development. The 21 kDa protein, which has pectinesterase inhibitor domain, and expansin-like B1 are implicated in the regulation of cell wall extension [35]. The above results indicate that, in comparison with 6-h treatment, a large number of genes were greatly up-regulated by 24-h H_2_O_2_ treatment. These genes are mainly associated with stress response, redox homeostasis, cell wall loosening and remodeling, auxin signalling pathway, ethylene and gibberellin signalling pathways, protein and lipid degradation, and cell differentiation and elongation. Notably, the genes coding for endoglucanase 17-like, expansin-like B1-like, MATE efflux family protein FRD3-like, and pectinesterase 2-like were highly up-regulated 2193-, 544-, 526-, and 182-fold, respectively, in HO24 vs. HO6, suggesting that cell wall loosening and remodeling and stress response were the profound molecular processes modulated by H_2_O_2_ from 6- to 24-h.

Six genes were down-regulated 8- to 134-fold in both HO24 vs. Wat24 and HO24 vs. HO6, including a cationic peroxidase 1-like, a germin-like protein subfamily 1 member 7-like, a CPRD49 (GDSL esterase/lipase), a 2-oxoglutarate/Fe(II)-dependent dioxygenase-like, a casparian strip membrane protein 3, a protease inhibitor, and a lipoxygenase gene (Additional file 7). The 2-oxoglutarate/Fe(II)-dependent dioxygenase belong to dioxygenase domain enzymes that is involved in the formation of plant hormones, such as ethylene, gibberellins, anthocyanidins and pigments such as flavones [35]. Lipoxygenases (LOXs) were involved in growth, development and oxidative stress [47] and were up-regulated in tissue cultures for adventitious rooting [48]. LOXs oxidize linolenic acid present in membranes, resulting in the lipoperoxidation of membranes [49, 50]. The down-regulation of this gene may alleviate the lipoperoxidation of membranes. Casparian strip membrane proteins (CASPLs) have recently been shown to form membrane scaffolds and direct the local modification of the cell wall in *Arabidopsis thaliana* [51].

### Genes involved in plant hormone signalling were significantly regulated during H_2_O_2_-induced adventitious rooting

Plant hormones are known to play vital roles in adventitious rooting; however, we do not known whether H_2_O_2_ promotes adventitious rooting by regulating the expression of plant hormone signalling-related genes. Thus, these genes were searched among all DEGs, and a total of 137 genes with both fold change > 2 and RPKM ≥ 5 were identified, including 21 ABC transport family members, 58 auxin pathway genes, 39 ethylene pathway genes, six cytokinin pathway genes, ten gibberellin-related genes, and three abscisic acid-related genes (Additional file 8 and Table 5).

**Table 5:** Plant hormone-related genes that were differentially regulated by H_2_O_2_ treatment

Products of genes	Total No.	HO6 vs.Wat6		HO24 vs.Wat24		HO24 vs.HO6	
		up	down	up	down	up	down
ABC transporter	21	8	0	3	0	4	9
ACO	6	0	0	2	0	1	3
ACS	2	0	0	1	0	0	1
AP2/ERF	26	2	7	3	6	15	5
Ethylene induced protein	5	0	0	1	1	0	4
ARF	7	2	0	1	0	0	5
AUX/IAA	10	0	2	1	1	1	8
PIN1	1	0	1	0	0	1	0
Auxin transporter	4	0	0	0	0	2	2
ABP1	1	0	0	0	1	1	0
Auxin induced protein	26	7	2	3	9	6	20
GH3	9	0	3	5	1	2	2
Cytokinin synthesis	5	1	0	1	3	1	3
Cytokinin induced protein	1	0	1	1	0	1	0
Gibberelin synthesis	7	1	0	0	1	0	7
Gibberelin receptor	1	0	0	0	0	1	0
Gibberelin induced protein	2	0	1	0	0	1	1
Abscisic acid receptor	2	0	1	0	2	0	0
Abscisic acid degradation	1	1	0	0	1	0	1
Total	137	22	18	22	26	37	71

Eight and three genes of ATP-binding cassette (ABC) transporter family members were up-regulated at 6 h and 24 h time points, respectively, but eight genes were down-regulated from 6 h to 24 h, suggesting that H_2_O_2_ increased the expression of ABC transport family genes. ABC transporters have been known to participate in the export or import of a wide variety of molecules for growth and development under abiotic stress [52] and to act as an auxin carrier complex in cellular auxin efflux and influx [53].

In the group of auxin-related genes, a total of 58 genes were identified, including 26 auxin-induced proteins, ten auxin response proteins (*AUX/IAA*), nine indole-3-acetic acid (IAA)-amido synthetase GH3s, seven auxin response factor (*ARF*), four auxin transporter-like protein (*LAX*), an auxin efflux carrier (*PIN1*), and an auxin-binding protein ABP19a (*ABP*) gene (Additional file 7). Of the auxin-induced protein genes, 14 auxin-induced protein 5NG4-like genes were up-regulated at 6 h but down-regulated at 24 h and from 6 h to 24 h. Among these genes, two auxin-induced protein PCNT115-like genes were highly up-regulated. The auxin-induced protein 5NG4-like proteins belong to the plant drug/metabolite exporter (DME) family that consists of a group of plant WAT1 (walls are thin1)-related proteins, which has been shown to be involved in secondary cell wall deposition [54]. The *AUX/IAAs* were down-regulated at 6 h and from 6 h to 24 h. The *ARFs* were up-regulated at 6 h and 24 h but down-regulated from 6 h to 24 h. The IAA-amido synthetase GH3 genes were down-regulated at 6 h but up-regulated at 24 h and from 6 h to 24 h. *PI*N1 and *LAX1* were up-regulated 6- to 20-fold from 6 h to 24 h. *ABP* was up-regulated 136-fold from 6 h to 24 h. The auxin-inducible Aux/IAA family proteins function as repressors of early auxin response genes at low auxin concentrations [55]. GH3s catalyse the synthesis of IAA-amino acid conjugates, providing a mechanism for the plant to cope with the presence of excess auxin [35]. PIN1 and LAX1 are involved in proton-driven auxin influx and mediate auxin gradient formation [56]. The ABP is probably an additional auxin receptor that mediates rapid cellular auxin effects through a non-transcriptional auxin response pathway [35]. These results suggest that H_2_O_2_ increased the expression of auxin transporter and receptor genes and down-regulated ARFs and AUX/IAAs from 6 h to 24 h after the treatment, resulting in the initiation of auxin signalling during H_2_O_2_-promoted adventitious rooting. This modulation mechanism of auxin signalling is similar to the IBA-induced process [30, 57] and was reported during adventitious rooting in petunia cuttings [5].

In the group of ethylene-related genes, 24 AP2-like ethylene-responsive transcription factor genes (AP2/ERFs), six 1-aminocyclopropane-1-carboxylate oxidase (ACO) genes, two 1-aminocyclopropane-1-carboxylate synthase (ACS) genes, and five ethylene-insensitive protein-like genes were identified. H_2_O_2_ treatment up-regulated the expression of ACO and ACS genes at 24 h but down-regulated their expression from 6 h to 24 h, indicating an increase in ethylene biosynthesis at 6 h compared with water treatment. Ethylene-insensitive protein 2 (EIN2), a central factor in signalling pathways regulated by ethylene, is involved in various processes including development, plant defences, senescence, nucleotide sugar flux, and tropisms [35]. The gene coding for ethylene-overproduction protein 1-like (ETO1), which functions as an essential regulator of the ethylene pathway by regulating the stability of ACS enzymes, was down-regulated from 6 h to 24 h [35]. Most AP2/ERF genes were down-regulated at 6 h but up-regulated from 6 h to 24 h. These genes may be involved in the regulation of gene expression by stress factors and by components of stress signal transduction pathways [35] and have also been shown during adventitious rooting in petunia [58]. Ethylene and its crosstalk with auxin have been showed to be required for adventitious root formation [58, 59]. These results suggest that the H_2_O_2_-induced process leading to adventitious rooting may modulate ethylene gene expression, and this mechanism has been revealed in IBA-induced adventitious rooting [30].

Six cytokinin-related DEGs were detected. Of these DEGs, a cytokinin-induced message gene that code for expansin-like B1 [35], was down-regulated 11-fold at 6 h but up-regulated 62-fold from 6 h to 24 h. A gene for cytokinin dehydrogenase 3 that degrades the phytohormone cytokinin [60], two genes for cytokinin hydroxylase-like that catalyses the biosynthesis of trans-zeatin in plants [35], and a cytokinin riboside 5′-monophosphate phosphoribohydrolase LOG1 that converts inactive cytokinin nucleotides to the biologically active free-base forms [35] were down-regulated from 6 h to 24 h. This result indicates that H_2_O_2_ treatment inhibited cytokinin activity via the regulation of cytokinin-related gene expression. The same result was shown in IBA-induced adventitious rooting [30].

Nine DEGs, including a gibberellic acid-stimulated protein 1, seven gibberellin oxidase genes, and a *GIR1* gene, were down-regulated from 6 h to 24 h, while a gibberellin receptor GID1B-like was up-regulated during this period. Both gibberellic acid-stimulated protein 1 and GIR1 are the GASA gibberellins-regulated cysteine-rich proteins that are involved in cell division and/or elongation and that respond to biotic and abiotic stresses [35, 61, 62]. The gibberellin oxidases are key enzymes in the biosynthesis of gibberellins and are involved in the production of bioactive GA for vegetative growth and development [35]. Similar to IBA treatment, H_2_O_2_ decreased the expression of the GA synthesis genes, leading to inhibition of GA synthesis [30].

In summary, the above results indicate that, during the process of H_2_O_2_-induced adventitious rooting, H_2_O_2_ initiated auxin and ethylene signalling and repressed cytokinin and gibberellins signalling for improving rooting.

### Transcription factor (TF)-encoding genes were significantly regulated during H_2_O_2_-induced adventitious rooting

We further analysed the TF-coding DEGs during H_2_O_2_-induced adventitious rooting. A total of 223 DEGs with both fold change > 2 and RPKM ≥ 5 were identified (Table 6 and Additional file 9). During the 6-h treatment, the genes coding for bHLH (9), MYB (8), zinc finger protein (ZF, 8), WRKY (7), NAC (4), MYB-like (7), homeobox-leucine zipper protein (HD-Zip, 5), Dof (3), ARF (2), and bZip (2) were highly up-regulated, while genes coding for AP2/ERF (7), LBD (5), and AUX/IAA (2) were down-regulated. Of those genes, the most highly up-regulated TF genes were AP2-like ethylene-responsive transcription factor At1g16060-like (7-fold) and MYB114 (5-fold), while the most highly down-regulated TF genes were LOB domain (LBD) -containing protein16 (21-fold), two ERF086-like (Vr42199 10-fold and Vr28889 12-fold), WRKY22-like (6-fold), and zinc finger protein ZAT11-like (4-fold). This result indicates that 6-h H_2_O_2_ treatment enhanced the expression of most of the TF genes except for most of the genes for AP2/ERFs and LBDs, which were decreased. During 24-h treatment, the genes coding for ZF (5), WRKY (6), and NAC (4) were up-regulated, while the genes coding for AP2/ERF (6) and bHLH (3) were down-regulated. Of those genes, ERF098-like (238-fold), zinc finger protein STOP1 homologue (89-fold), WRKY36 (4-fold), and WRKY31-like (4-fold) were the most highly up-regulated TF genes, while the most down-regulated TF gene was transcription factor ORG2-like (9-fold). Clearly, the degree of up-regulation of the TF genes was higher than that of down-regulation, indicating that 24-h H_2_O_2_ treatment highly enhanced the expression of certain TF genes.

**Table 6:** Transcription factor-coding genes that were differentially regulated by H_2_O_2_ treatment

TF families	Total No.	HO6 vs.Wat6		HO24 vs.Wat24		HO24 vs.HO6	
		up	down	up	down	up	down
bHLH	29	9	0	0	3	3	21
AP2/ERF	26	2	7	3	6	14	7
MYB	25	8	3	2	3	9	9
ZF	24	8	2	5	2	10	6
WRKY	17	7	1	6	1	6	6
NAC	16	4	0	4	2	5	7
MYB-like	11	7	0	0	0	3	3
Homeobox	10	0	0	0	1	1	9
AUX/IAA	10	0	2	1	1	1	8
HSF	8	0	0	0	0	3	5
Nuclear TF	8	2	0	0	0	0	7
ARF	7	2	0	1	0	0	5
HD-Zip	6	5	0	0	0	0	2
LBD	6	0	5	0	1	5	0
Dof	5	3	0	0	0	2	1
bZip	4	2	0	1	1	1	2
GATA	2	0	0	0	0	2	0
Others	9	4	1	0	0	1	3
Total	223	63	21	23	21	66	101

Comparing HO24 with HO6, the TF genes for AP2/ERF (14), MYB (9), ZF (10), LBD (5), WRKY (6), and NAC (5) were up-regulated, of which the genes for ERF LEP-like, ERF086-like, LBD 16-like, ERF098-like (Vr113471 and Vr70962), ERF088-like, WRKY31-like, AP2/ERF AIL6-like, zinc finger protein STOP1 homolog, WRKY36, zinc finger protein ZAT11-like, MYB-like, bHLH135-like, LOB 33-like, NAC 100-like (Vr54310 and Vr44686), bHLH93-like, MADS-box transcription factor 1-like, HSF B1, MYB134, LOB 13-like, MYB114, HSF B-3-like, and MYB114 were up-regulated 4- to 202-fold. The genes for bHLH (21), homeobox (9), MYB (9), AUX/IAA (8), nuclear TF (7), NAC (7), HSF (5), AP2/ERF (7), WRKY (6), and ZF (6) were down-regulated, of which the genes for MYB60, homeobox-leucine zipper protein HOX27-like, ERF034-like, MYB4-like, bHLH126-like, ERF 15-like, zinc finger CCCH domain-containing protein 15-like, NAC 43-like, MYB86-like, bHLH18-like, zinc finger protein CONSTANS-LIKE 15-like, HSF A-6b-like, transcription factor PIF4-like, zinc finger protein MAGPIE-like, bHLH137-like, transcription factor UNE10-like, HSF C-1-like, transcription factor BEE 3-like (Vr56647 and Vr31512), ERF SHINE 3-like, and AP2/ERF At1g16060-like were down-regulated 4- to 14-fold. This result indicates that most of the TF genes were down-regulated from 6 h to 24 h except for AP2/ERF, ZF, and LBD family genes; moreover, the degree of up-regulation of these genes is far greater than that of down-regulation.

Notably, Aux/IAA proteins are short-lived transcriptional factors that function as repressors of early auxin response genes at low auxin concentrations [35]. H_2_O_2_ treatment down-regulated the *AUX/IAA* genes but up-regulated the *ARF* genes, suggesting the involvement of H_2_O_2_ in the auxin signalling, which has been shown to be a key regulator of adventitious rooting [1]. Furthermore, LBD family genes are up-regulated by auxin [30, 63, 64] and have been found to act early in auxin signalling and to regulate adventitious rooting [5]. Of those genes, *LOB16* and *LOB29* were up-regulated 16- to 32-fold by IBA treatment [30]. In this study, five LBD family genes were up-regulated 4- to 31-fold by H_2_O_2_ treatment from 6 h to 24 h, of which *LOB16* was up-regulated 31-fold. Most of the AP2/ERF family genes were down-regulated at both 6 h and 24 h but up-regulated from 6 h to 24 h. For example, genes for ERF LEP and ERF086-like were up-regulated by 202-fold and 60-fold, respectively. These two genes have been shown to act as cell division-promoting factors involved in various processes of plant developments such as leaf blade differentiation and inflorescence branching [65]. ERF086 is also involved in the control of cell division patterns during early lateral root primordium development [65]. Although most of the AP2/ERF family genes were down-regulated at both 6 h and 24 h, the ERF At1g16060-like and ERF098-like were up-regulated by 7- and 238-fold at 6 h and 24 h, respectively. These two genes have been known to be involved in the regulation of gene expression by stress factors and by components of stress signal transduction pathways [35]. These results imply that H_2_O_2_ treatment initiated the ethylene signalling pathway, leading to adventitious rooting via up-regulating both the expression of stress responsive ERF genes at 6 h and 24 h and cell developmental ERF genes from 6 h to 24 h. Recently, a number of studies have reported that the AP2/ERF family genes were the most highly regulated TFs [66] and were key endogenous regulators of adventitious rooting in petunia [58] and poplar [67] and IBA-induced adventitious rooting in mung bean [30]. In addition, the WRKY and NAC family genes were highly up-regulated at 6 h and 24 h. These two families of genes have been known to be involved in plant defence responses. NAC proteins are also involved in developmental processes, including the formation of the shoot apical meristem, floral organs and lateral shoots, as well as in plant hormonal control [35]. Some of NAC proteins, such as transcription factor JUNGBRUNNEN 1-like, are involved in modulating cellular H_2_O_2_ levels and enhancing tolerance to various abiotic stresses through the regulation of DREB2A [68].

### Heat shock proteins (HSPs)-coding genes were significantly regulated by H_2_O_2_ treatment

Interestingly, we found that a large number of genes coding for heat shock proteins (HSPs) and heat stress transcription factors (HSFs) were significantly up-regulated during H_2_O_2_ treatment. Therefore, we screened the genes with both fold change > 2 and RPKM ≥ 5, and 29 HSPs and 5 HSFs were identified (Table 7). Amongst these genes, 25 HSP genes were up-regulated 2- to 98-fold at 6 h, 20 HSP genes were up-regulated 2- to 94-fold at 24 h, and 29 HSP genes were up-regulated 2- to 4-fold from 6 h to 24 h. Heat shock proteins are known to act as stress proteins, and their up-regulation is described more generally as part of the stress response [69, 70]. Many researchers have reported that this family of proteins was produced by cells in response to exposure to stressful conditions, including heat shock, cold, UV light, nitrogen deficiency, water deprivation, and wounding [69, 70], as well as exogenously applied H_2_O_2_ [14]. Many HSP members function as intra-cellular chaperones that can protect other proteins against stress-induced denaturation and aggregation by stabilizing new proteins to ensure correct folding or by helping to refold proteins that were damaged by stress [69, 70]. Clearly, our result indicates that the up-regulation of HSP genes and HSF genes is an important mechanism under H_2_O_2_-induced adventitious rooting in mung bean seedling.

**Table 7:** HSP coding genes that were differentially up-regulated by H_2_O_2_ treatment

Gene_ID	Fold-change			Nr reference	description
	HO6 vs. Wat6	HO24 vs. Wat24	HO24 vs. HO6		
Vr32973	NA	30.93	2.64	ref|XP_004504869.1|	18.2 kDa class I heat shock protein-like
Vr85607	97.85	24.01	2.21	sp|P30236.1|	22.0 kDa class IV heat shock protein
Vr45895	54.93	6.24	3.64	ref|XP_003529343.1|	17.5 kDa class I heat shock protein-like
Vr37499	47.63	94.22	3.96	ref|XP_003550253.1|	17.9 kDa class II heat shock protein-like
Vr62530	32.27	55.87	3.12	ref|XP_003618790.1|	8.2 kDa class I heat shock protein
Vr54154	25.97	63.19	3.96	ref|XP_003550253.1|	17.9 kDa class II heat shock protein-like
Vr46055	21.50		3.30	ref|XP_003538574.1|	small heat shock protein
Vr57261	19.44	3.55		ref|XP_003523325.1|	small heat shock protein
Vr49859	19.37	9.33	3.67	ref|XP_004495437.1|	22.7 kDa class IV heat shock protein-like
Vr42894	14.44		3.87	ref|XP_003549537.1|	heat shock 70 kDa protein-like
Vr26187	12.65	9.52	2.02	ref|XP_002512741.1|	heat shock protein
Vr40796	12.10		4.79	ref|XP_003549537.1|	heat shock 70 kDa protein-like
Vr67841	11.31	7.23	2.73	ref|XP_002512742.1|	heat shock protein
Vr43715	9.29	2.87	3.62	ref|XP_003522277.1|	17.9 kDa class II heat shock protein-like
Vr44383	7.77	2.58	3.32	ref|XP_003519372.1|	18.2 kDa class I heat shock protein
Vr45038	7.27			gb|ADU55794.1|	HSP18.1B
Vr53943	6.18	33.06	3.24	ref|NP_001234130.1|	cytosolic class II small heat shock protein HCT2
Vr34694	4.57	2.35	4.06	ref|XP_004516872.1|	heat shock protein 83-like
Vr38900	4.35	2.00	4.49	ref|XP_003542731.1|	18.5 kDa class I heat shock protein-like
Vr36118	4.13	3.18	4.01	ref|NP_001235177.1|	18.5 kDa class I heat shock protein
Vr43694	3.39		2.47	gb|AEY83985.1|	heat shock protein 101 Kda
Vr55757	3.25	4.15	3.48	ref|XP_003517579.1|	heat shock cognate 70 kDa protein 4-like
Vr50664	2.62	2.38	2.56	ref|XP_003552695.1|	heat shock cognate 70 kDa protein-like
Vr12901	2.23	2.25	2.69	ref|XP_003552695.1|	heat shock cognate 70 kDa protein-like
Vr44363	2.22	2.38		ref|XP_003528707.1|	17.4 kDa class III heat shock protein-like
Vr15042			2.28	ref|XP_002263599.1|	heat shock cognate 70 kDa protein isoform 1
Vr31797			2.23	emb|CAA47345.1|	70 kDa heat shock protein
Vr36475			2.12	ref|XP_003544594.1|	heat shock cognate protein 80-like
Vr37094			2.02	gb|AAS57912.1|	70 kDa heat shock cognate protein 1
Vr28793	4.33	3.29		gb|AFQ93676.1|	heat shock transcription factor HSFA2
Vr25153	47.48			ref|XP_003536914.1|	heat stress transcription factor A-6b-like
Vr13888			4.61	emb|CAA87077.1|	heat shock transcription factor 34
Vr26860			4.20	ref|XP_003521171.1|	heat stress transcription factor B-3-like
Vr27046			2.35	ref|XP_003546045.1|	heat stress transcription factor A-4a-like

### Validation of gene expression by qRT-PCR

**Fig. 5:**
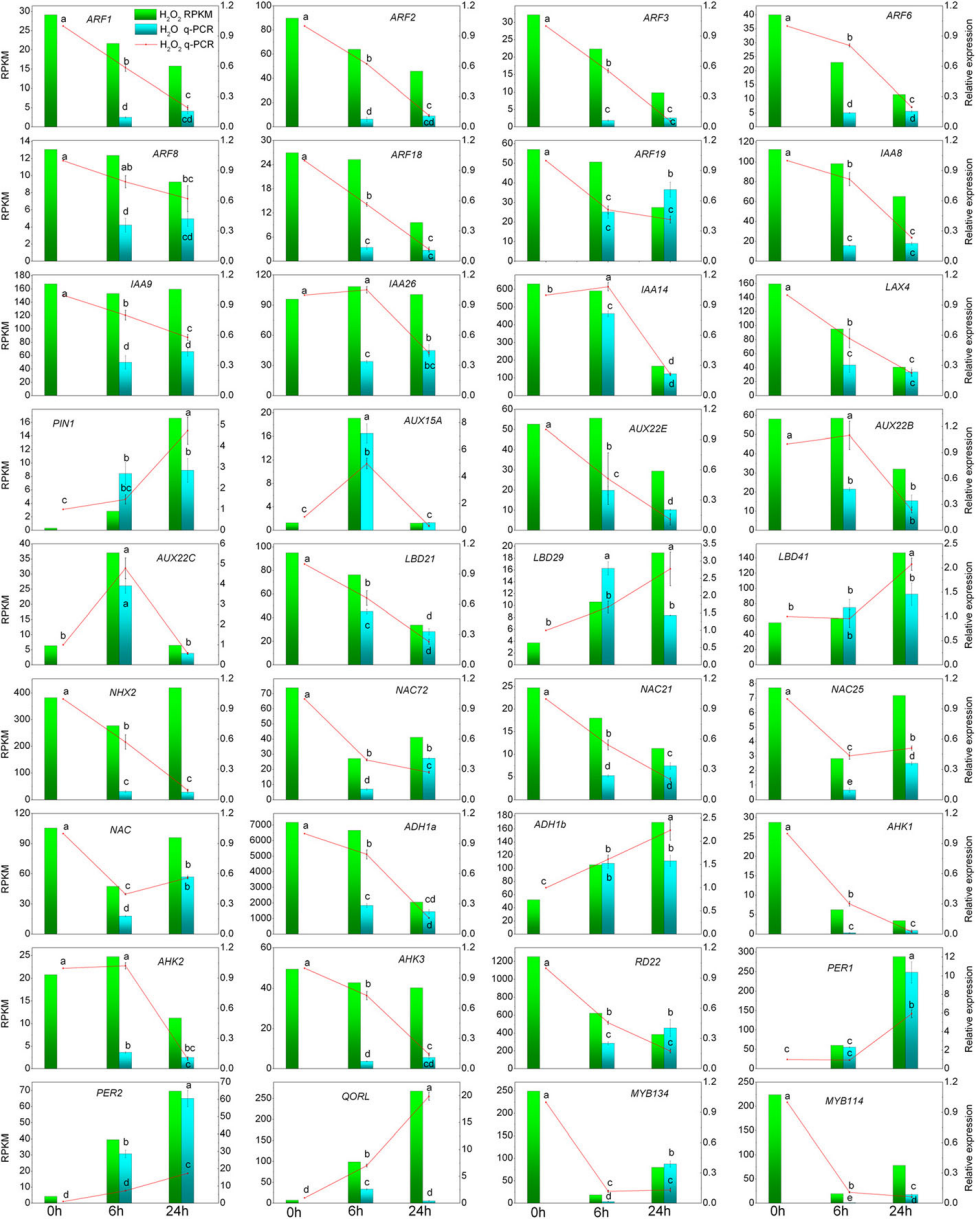
Validation for the RNA-Seq data using qRT-PCR. The qRT-PCR data were compared with RNA-Seq data and the Con, Wat 6, and Wat 24 data were from our previous study [29]. *Bars* represent the mean (±SE) of three replicates. Different letters (a, b, and c) represent statistically significant differences (*P* <0.01) among the qRT-PCR data, which were analysed using Student’s *t*-test

The RPKM values of the DEGs obtained from the transcriptome were validated using real-time quantitative PCR (qRT-PCR). For qRT-PCR, we selected a total of 36 interesting DEGs; the results are shown in Fig. 5. Detailed information regarding these genes is presented in Additional file 10. We selected the housekeeping gene *CPY20* as an internal reference gene for qRT-PCR measurement. The expression levels of most of the genes, as measured by qRT-PCR, showed a strong correlation to the RNA-Seq data (more than 83% of the dataset had a correlation coefficient r > 0.9). The qRT-PCR results showed that the expression levels of 26 genes, including *ARF1*, *ARF2*, *ARF3*, *ARF6*, *ARF8*, *ARF18*, *ARF19*, *IAA8*, *IAA9*, *IAA14*, *IAA26*, *LAX4*, *AUX22E*, *AUX22B*, *LBD24*, *NHX2*, *NAC*, *NAC21*, *NAC25*, *NAC72*, *RD22*, *AHK1*(*Arabidopsis histidine kinase 1*), *AHK2*, *AHK3*, *MYB134*, and *MYB114*, significantly decreased at 6 h and 24 h in both the water and H_2_O_2_ treatments, but H_2_O_2_ treatments significantly up-regulated their expression at 6 h compared with the water controls. The expression levels of *PIN1*, *AUX15A*, *LBD29*, *LBD41, ADH1b*, *QORL* (*quinone oxidoreductase-like protein*), *PER1* (*cationic peroxidase 1*), and *PER2* significantly increased at both 6 h and 24 h. Compared with the water controls, H_2_O_2_ treatments significantly up-regulated the expression of *PIN1*, *AUX15A*, *LBD29*, *LBD41*, and *ADH1b* at 24 h; up-regulated the expression of *QORL* at both 6 h and 24 h, but down-regulated the expression of *PER1* and *PER2* at both 6 h and 24 h. The expression of *AUX15A* and *AUX22c* significantly increased at 6 h and then decreased at 24 h. This result is fully consistent with the DEGs analysis from the RNA-Seq data, and further indicates the roles of H_2_O_2_ in regulating genes expression.

The genes involved in the auxin signalling pathway are required for the formation of adventitious roots. The auxin efflux carrier PIN family members and auxin influx carrier AUX family members mediate auxin polar transport and control auxin distribution to establish and maintain auxin concentration gradients for various developmental processes [71] including adventitious rooting [57]. The ARFs [72], GH3-like proteins [73], and LBDs [48, 74, 75] have also been shown to be essential for adventitious root formation. NAC proteins are involved in plant developmental processes [76, 77] and defence responses [78]. For example, NAC21 mediates auxin signalling to promote lateral root development [79]. AHK1 functions as an osmosensor histidine kinase and is required for the regulation of desiccation processes [80]. AHK2 acts as a cytokinin receptor and, together with AHK3, functions as a redundant negative regulator of drought and salt stress responses and abscisic acid signalling [81]. These proteins were shown to be involved in the water stress response during the early vegetative stages of plant growth and regulation of meristem development [82]. *Arabidopsis* mutants lacking AHK2, AHK3, and AHK4 exhibited enhanced adventitious root growth [83]. *ADH1* (*Alcohol dehydrogenase 1*) and *NHX2* (*Na* ^*+*^ */H* ^*+*^ *exchanger protein*) are involved in the osmotic stress response [84]. Dehydration-responsive protein RD22 was up-regulated by dehydration, salt stress and abscisic acid and can enhance drought tolerance [85]. The *Arabidopsis* homologue of quinone oxidoreductase-like protein (QORL) belongs to chloroplastic NADPH-dependent alkenal/one oxidoreductase (AOR) which contributes to detoxifying lipid peroxide-derived reactive carbonyls (RCs) produced under oxidative stress and to protect respiration and growth [86]. PERs are involved in the removal of H_2_O_2_, oxidation of toxic reductants, biosynthesis and degradation of lignin, suberization, auxin catabolism, and responses to environmental stresses, such as wounding, pathogen attack and oxidative stress. Briefly, these q-PCR results confirmed that H_2_O_2_-priming up-regulated the expression of several auxin signalling-related genes, such as *ARFs*, *AUX/IAAs*, and *PINs*, and several genes from the *LOB* and *MYB* transcription factor families, particularly promoting the expression of various stress response- and oxidative-related genes, such as *PERs* and *QORL*, to further increase the stress tolerance of the cells.

In summary, all the above results indicate that a majority of most highly up- or down-regulated genes were similar during both H_2_O_2_- and IBA-induced adventitious rooting, suggesting the similar regulation pattern of gene expression in the processes [30]. This supports the concept that H_2_O_2_ is an important component of auxin-mediated adventitious root formation in cuttings [5]. For example, both H_2_O_2_ and IBA treatments up-regulated the genes coding for auxin signalling pathway proteins GH3s, ABP, and auxin-induced proteins, whereas down-regulated Aux/IAA and ARFs genes; up-regulated genes coding for ethylene signalling pathway proteins AP2/ERFs, ACS, and ACO, while down-regulated some members of AP2/ERFs; up-regulated cytokinin-induced message genes but down-regulated cytokinin synthesis genes; up-regulated the cell wall related expansins and polygalacturonse genes while down-regulated CASPL genes; up-regulated the most of LOBs and WRKYs transcription factor genes but down-regulated the most of bHLHs and MYBs genes; up-regulated GST, PODs, and polyphenol oxidase genes that are related to cell redox homeostasis but down-regulated some members of POD genes; up-regulated GDSL esterases and sugar transporter genes involved in metabolism but down-regulated LOX genes. However, some significant differences on gene expression levels were also observed between H_2_O_2_ and IBA treatments. For example, major of the most highly regulated genes by H_2_O_2_ were those involved in stress response, for IBA, were those involved in auxin signalling, and ethylene signaling pathways, as well as LOB family and meristems and primordial development-related. In addition, a lot of genes involved in secondary metabolism and flavonoid biosynthesis were down-regulated by IBA. Many of small HSPs and HSFs genes were up-regulated by H_2_O_2_. In short, IBA application primarily initiated the auxin signalling processes, whereas, H_2_O_2_-priming initially started the stress response processes, further leading to adventitious rooting in mung bean seedlings [30].

## Conclusions

It is well known that H_2_O_2_ functions as an important regulator molecule that is involved in many signalling pathways in plant cells. Exogenous application of H_2_O_2_ at an appropriate level has been demonstrated to improve cell tolerance to abiotic and biotic stresses. Low concentration application of H_2_O_2_ also acts as a promoter of adventitious root formation. The present study provides a transcriptome insight into the molecular basis of specific responses to H_2_O_2_ priming under adventitious rooting in mung bean. H_2_O_2_ treatment highly increased the number of reads, the ratio of expressed genes, and the abundance of gene expression. In terms of the time duration, H_2_O_2_ strongly influenced the cell metabolism and functions by regulating gene expression, particularly up-regulating gene expression at 6-h time point, which is the induction phase for adventitious rooting in mung bean. The Gene Ontology (GO) classification, Kyoto Encyclopedia of Genes and Genomes (KEGG) pathway enrichment, and differentially expression genes (DEGs) profiling results revealed that H_2_O_2_ triggers adventitious rooting through the regulation of many cellular processes, including the functions associated with stress response, cell redox homeostasis and oxidative stress response, secondary metabolism and flavonoid synthesis, cell wall loosening and modification, nutrients and energy metabolic processes, cellular component movement, transcription, DNA synthesis, cell cycle, and transcription factors (TFs), as well as plant hormone signalling pathways. In some ways, the gene expression profile that specifically responds to H_2_O_2_ during adventitious rooting in mung bean was similar to that in response to IBA. Last, this study provides a list of candidate genes that specifically respond to H_2_O_2_ priming in plants.

**Fig. 6:**
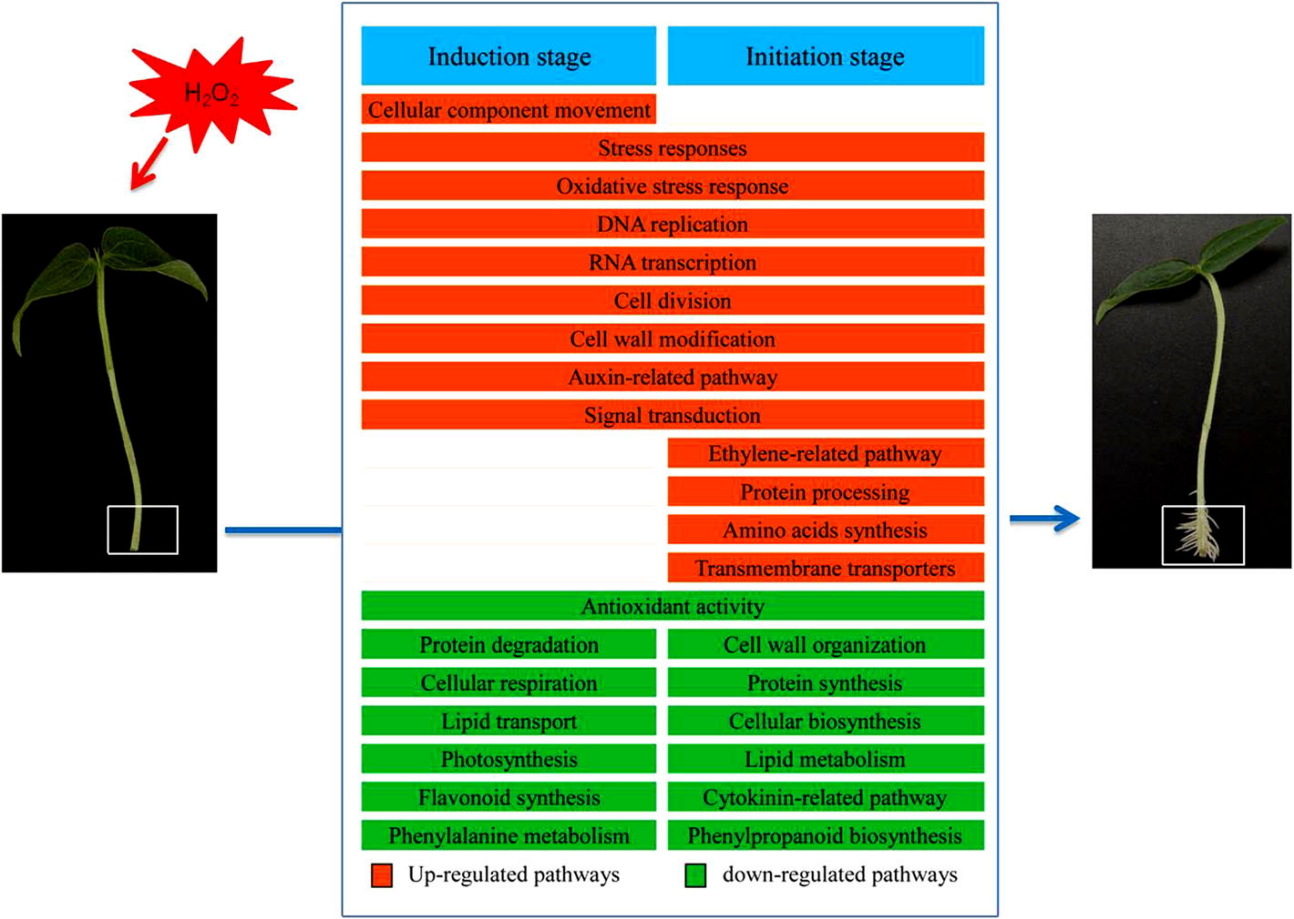
A proposed model of important pathways that were highly regulated by H_2_O_2_-priming illustrates the molecular respects during the induction and initiation stages of adventitious roots in mung bean seedlings. The most highly regulated genes under each pathway are listed in Fig. 4. and Additional files 2, 3, 4, and 5

By integrating the results of GO and KO enrichment and analyses of most highly differentially expressed genes in response to H_2_O_2_, we proposed a model including the most highly regulated pathways by H_2_O_2_-priming that might be involved in the induction and initiation of adventitious roots in mung bean seedlings (Fig. 6).

## Methods

### Plant material and culture conditions

After mung bean [*Vigna radiata* (L.) R. Wilczek] seeds were surface-sterilized in a 6% NaClO solution for 15 min and rinsed three times in sterile distilled water, they were covered with a thin layer of sterilized perlite and germinated in Petri dishes in a growth chamber at 25 ± 1 °C for 36 h in the dark and then at 25 ± 1 °C with a 14-h light/10-h dark photoperiod under white fluorescent lamps (PAR of 100 μM m^−2^ s^−1^). The five-day seedlings that were 5 cm in height were used as experimental material. After the primary roots were removed from the bases of the hypocotyls, the resulting seedlings were incubated in 50-mL beakers (10 per beaker), each containing 40 mL of sterilized distilled water or 10 mM H_2_O_2_ under the same aseptic conditions as the seedling culture. To evaluate the gene expression profile during adventitious rooting, the basal part (5 mm in length) of each hypocotyl, where the adventitious roots would form, was harvested separately at the 0, 6, and 24 h incubation time points and used as samples for RNA extraction. Each sample contained ten stem cutting bases in three biological replicates in parallel. The samples were separately marked as Con, Wat6, HO6, Wat24, and HO24, immediately frozen in liquid nitrogen and stored at −80 °C until further analysis.

### Total RNA extraction, cDNA library construction and Illumina RNA-Seq

For total RNA extraction, 50 mg of the samples was fully ground in liquid nitrogen, and total RNA extraction was performed using a plant total RNA Kit according to the manufacturer’s protocol (kit SK8631, Sangon, Shanghai, China). The resulting total RNA was dissolved in 50 μL of RNase-free water, and stored at −80 °C. The RNA integrity was quantified with RNA integrity number (RIN) values of 8.1-9.9 using a 2100 Bioanalyzer (Agilent Technologies, Santa Clara, CA, USA), and RNA concentration was determined using a NanoDrop ND-1000 Spectrophotometer.

Equal amounts of total RNA from each sample were used to purify the Poly (A) mRNAs using Oligo(dT) 25 beads (Invitrogen) according to the manufacturer’s instructions. The Fragment Mix reactive system was used to fragment the purified mRNA at 94 °C for 4 min. The first-strand cDNA was synthesized from fragmented mRNA templates using Superscript II reverse transcriptase (Invitrogen), random hexamer primers, and First Strand Master Mix at 25 °C for 10 min, 42 °C for 50 min, and 70 °C for 15 min, with a final hold at 4 °C. The RNA template was then removed and second-strand cDNA was synthesized using Second Strand Master Mix (Invitrogen). After the double-strand cDNA was synthesized, it was purified with Agencourt AMPure XP Beads (Agencourt), and the 3′ ends were repaired using End Repair Control, followed by purification with AMPure XP beads. Subsequently, an A-base was added to the blunt end fragments using Klenow exo (M0212L, NEB) to perform the adenylation of the 3′ ends of the cDNA fragments. Thereafter, Illumina indexing adapters were ligated to the cDNA fragments using T4 Ligase (Fermentas) according to the standard protocol. After the ligated cDNAs were purified twice using AMPure XP Beads, they were enriched and amplified using selective PCR according to the following procedure: 98 °C for 30 s; 15 cycles of 98 °C for 10 s, 60 °C for 30 s, 72 °C for 30 s, and 72 °C for 5 min; holding at 4 °C, followed by purification with AMPure XP beads. The quality and concentration of the cDNA library were confirmed using an Agilent 2100 Bioanalyzer and Qubit 2.0 (Life Technologies). The resulting cDNA libraries were then paired-end sequencing on an Illumina HiSeq 2000 system and performed at Sangon Biotech Co., Ltd. (Shanghai, China). The sequencing quality was checked using FastQC V0.10.1 with an ASCII Q-score offset of 33.

### De novo assembly and sequence clustering

For de novo assembly, the adaptor sequences, low quality reads, and reads with unknown sequences > 5% were removed from the raw sequence, and then the resulting high quality sequence reads were de novo assembled using the Trinity paired-end assembly method [31]. The assembled sequences were clustered with Chrysalis. The longest sequences that could not be extended on either end within each clustered loci were obtained and defined as unigenes [29]. Finally, similarity alignment of the resulting unigenes were performed against the public protein and nucleotide sequence databases NR, SWISS-PROT, TrEMBL, Pfam, and CDD with similarity set at >30% and an E-value ≤ 1e-5 using BLASTx locally installed BLAST+ v2.2.27 software [87] and MEGABLAST, respectively. BLAST annotations were filtered using either subject or query coverage (>30%) and sequence identity (>50% for MEGABLAST and >30% for BLASTx). The results that presented the best alignment were used to identify the sequence direction and to predict the coding regions using BLASTx searches against protein databases, with the priority order of NR, SWISS-PROT, KEGG and KOG if conflicting results were obtained. The ESTScan software [88] was used to analyse the unigenes that did not align to any of the above databases. The assembled unigenes were deposited in the Transcriptome Shotgun Assembly Sequence Database (http://www.ncbi.nih.gov/genbank/tsa.html) at DDBJ/EMBL/GenBank under the sequence read archive SRR 1653637 and the accession numbers GBXO01000001-GBXO01078617.

### GO and KEGG pathway enrichment of the unigenes

To understand which functional categories of DEGs were modulated during H_2_O_2_-induced adventitious rooting in mung bean seedlings, the unigenes were further aligned against the KOG (Clusters of Orthologous Groups for eukaryotic complete genomes) and KEGG (Kyoto Encyclopedia of Genes and Genomes) pathway annotations using BLASTALL and KEGG Automatic Annotation Server (KAAS) software with an E-value ≤ 1e-5. The resulting blast hits were processed using Blast2GO software (version 2.3.5) [31] with an E-value threshold of 1e-5 to retrieve associated Gene Ontology (GO) terms, and WEGO software was used for achievement of GO classification [31]. To further enrich the pathway annotations, unigenes were submitted to the KAAS [33], and the single-directional best-hit information method was selected. To identify the enriched pathways, the phyper test was used to measure the relative coverage of the annotated KEGG orthologous groups of a pathway against the transcriptome background, and the pathways with a *p*-value ≤ 0.05 were classified as enriched. The difference analysis between a pair samples was performed between HO6 and Wat6, HO24 and Wat24, and HO24 and HO6 and was represented as HO6 vs. Wat6, HO24 vs. Wat24, and HO24 vs. HO6, respectively.

### Unigene expression analysis and DEG confirmation

To calculate unigene expression levels, the unigene sequences were mapped to the reference sequences [29, 30, 89]. The clean reads were mapped back to the assembled unigenes using Bowtie 2 in the end-to-end alignment mode, and the number of clean reads that were mapped to each unigene was calculated and then normalized to RPKM (reads per kb per million reads) using ERANGE3.1 software [90]. The DEGseq R package was used to analyse the unigene expression levels [38] with the MARS (MA-plot-based method with Random Sampling) model. To further know the DEGs that were specifically regulated during H_2_O_2_-induced adventitious rooting, we screened the DEGs and their expression levels between HO6 and Wat6, HO24 and Wat24, and HO24 and HO6. The DEGs between each pair of samples were screened using the Audic-Claverie algorithm [91] with an FDR threshold of ≤0.001 and an absolute value of log2 ≥ 1, and the Benjamini-Hochberg correction was used to perform multiple test corrections for the *p*-value and FDR [92].

### Quantitative reverse transcription PCR (qRT-PCR) validation

To validate the results from RNA-Seq, qRT-PCR was performed. For q-PCR, each of three biological replicates (*n* = 10) same as those subjected to Illumina RNA-Seq were used for total RNA extraction. Total RNA was extracted using TRIzol reagent (Invitrogen, Carlsbad, CA, USA) according to the manufacturer’s protocol. RNA integrity was examined with an Agilent Bioanalyzer 2100 (Agilent Technologies). In brief, cDNA samples preparation was performed as described in our previous study [30]. In this experiment, 36 genes that had been shown to be involved in auxin-related development and the stress response were selected for q-PCR analysis. The genes *CPY20*, *eIF5A*, and *ACTIN* (Actin-related protein 4) were selected as internal reference genes according to the RNA-Seq results and a previous report [93]. The gene-specific primer pairs were designed using Primer Premier 5.0 software (Applied Biosystems, Foster City, CA, USA) according to the sequences from RNA-Seq (Additional file 10). Real-time PCR was run using a LightCycler 480 II (Roche Applied Science) and ABI SYBR Green PCR Master Mix (ABI, Foster, USA). The PCR cycling conditions were as follows: 95 °C for 3 min, 40 cycles at 95 °C for 15 s, and 60 °C for 40 s. For negative controls, ‘No cDNA’ samples (water) and ‘no RT’ samples were included. All reactions were performed in triplicate. To evaluate the primer specificity for each primer set, melting curve analysis was conducted to verify the presence of a single melting peak. All q-PCR reactions were carried out with three independent biological replicates. The relative expression levels of the selected genes were calculated in relation to the reference gene using the comparative threshold cycle method with the delta-delta Ct method [94]. Statistical analyses were conducted with Student’s t-tests at the *P* < 0.05 level of significance.

## Additional files

Additional file 1: List of the most significantly up- and down-regulated GOs. (XLSX 77 kb)
Additional file 2: List of up-regulated genes in the 6-h hydrogen peroxide treatment. (XLSX 31 kb) (XLSX 62 kb)
Additional file 3: List of down-regulated genes in the 6-h hydrogen peroxide treatment. (XLSX 31 kb)
Additional file 4: List of up-regulated genes in the 24-h hydrogen peroxide treatment. (XLSX 41 kb)
Additional file 5: List of down-regulated genes in the 24-h hydrogen peroxide treatment. (XLSX 31 kb)
Additional file 6: List of up-regulated genes between the 24-h hydrogen peroxide treatment and the 6-h treatment. (XLSX 95 kb)
Additional file 7: List of down-regulated genes between the 24-h hydrogen peroxide treatment and the 6-h treatment. (XLSX 31 kb)
Additional file 8: List of differential expressed genes associated with plant hormone signalling. (XLSX 24 kb)
Additional file 9: List of differential expressed TFs. (XLSX 32 kb)
Additional file 10: List of the genes and primers selected for q-PCR validation. (PDF 106 kb)

## Abbreviations

ABCB/PGP: ATP binding cassette-type B/ P-Glycoproteins
ACC: 1-Aminocyclopropane-1-carboxylic acid
ACO: 1-Aminocyclopropane-1-carboxylic acid oxidase
ACS: 1-Aminocyclopropane-1-carboxylic acid synthase
AHK: Arabidopsis histidine kinase
AOX: Alternative oxidase
AP2/ERF: Apetala2/Ethylene response factor
APX: Ascorbate peroxidase
ARF: Auxin response factor
ARL1: Adventitious rootiless1
AUX1/LAX: Auxin/IAA
bHLH: Basic/helix-loop-helix
bZIP: basic leucine zipper
CASPL: Casparian strip membrane protein-like
CAT: Catalase
CHS: Chalcone synthase
CRL1: Crown rootless1
DEGs: Differentially expressed genes
Dof: Dof zinc finger protein
EIN: Ethylene-insensitive protein
ETO: Ethylene-overproduction protein
GATA: GATA transcription factor
GH: Glycoside hydrolase
GH3: Gretchen hagen 3
GO: Gene ontology
GPX: Glutathione peroxidase
GR: Glutathione reductase
GST: Glutathione S-transferase
HD-Zip: Homeobox-leucine zipper protein
HSFs: Heat shock transcription factors
HSPs: Heat shock proteins
IBA: Indole-3-butyric acid
JA: Jasmonic acid
KAAS: Automatic annotation server
KEGG: Kyoto encyclopedia of genes and genomes
KOG: Clusters of orthologous groups for eukaryotic complete genomes
LBD16: Lateral organ boundaries-domain
LEA: Late embryogenesis abundant
LOB-domain: Lateral organ boundaries-domain
LOXs: Lipoxygenases
LRR: Leucine-rich repeat
MAPK: Mitogen-activated protein kinase
NAC: NAM, ATAF, and CUC2
NAM: No apical meristem
NDPK: Nucleotide diphosphate kinase
NR: NCBI non-redundant protein
PCD: Programmed cell death
PER: Peroxidase
PIN: PIN-formed
PYR: Pyrabactin resistance
QC: Quiescent center
QORL: Quinone oxidoreductase-like protein
qRT-PCR: Real-time quantitative polymerase chain reaction
RAM: Root apical meristem
RNA-seq: RNA-sequencing
ROS: Reactive oxygen species
RPKM: Reads per kb per million reads
RTCS: Rootless concerning crown and seminal roots
SAM: S-adenosylmethionine
SCR: Scarecrow
TFs: Transcription factors
WRKY: WRKYGQK domain protein
ZF: Zinc finger protein
