# Supplementary material for: Transcriptomic profiling provides molecular insights into hydrogen peroxide-induced adventitious rooting in mung bean seedlings
Source: BMC Genomics. 2017 Feb 17;18:188. doi: 10.1186/s12864-017-3576-y (PMC5316208; doi:10.1186/s12864-017-3576-y)
Supplement: Additional file 10: — List of the genes and primers selected for q-PCR validation. (PDF 106 kb) [file 12864_2017_3576_MOESM10_ESM.pdf]

Additional file 10 List of the genes selected for q-PCR validation

Gene ID	Functional description	Swissprot ID	Bitscore	E-value	RPKM		
					Con	Wat6	Wat24
Vr37254	Auxin response factor 2, ARF2	sp|Q94JM3	952	0	89.852	37.954	50.760
Vr35374	Auxin response factor 18, ARF18	sp|Q653H7	816	0	26.952	16.698	14.999
Vr35831	Auxin response factor 1, ARF1	sp|Q8L7G0	885	0	29.128	15.349	16.768
Vr29470	Auxin response factor 3, ARF3	sp|O23661	450	1E-142	32.138	10.622	12.376
Vr13756	Auxin response factor 6, ARF6	sp|Q9ZTX8	376	2E-114	40.028	15.658	13.549
Vr36957	Auxin response factor 19, ARF19	sp|Q8RYC8	734	0	57.040	24.402	29.694
Vr24483	Auxin response factor 8, ARF8	sp|Q9FGV1	278	3E-82	13.053	8.386	8.696
Vr23260	Auxin transporter-like protein 4	sp|Q8L884	851	0	159.520	73.881	49.185
Vr34322	LOB domain-containing protein 41, LBD41	sp|Q9M886	185	3E-53	55.243	121.677	134.119
Vr39013	LOB domain-containing protein 21, LBD21	sp|Q9SRL8	188	1E-54	94.987	84.197	46.743
Vr40540	LOB domain-containing protein 29, LBD29	sp|Q9M2J7	186	3E-54	3.714	26.745	12.554
Vr38129	Sodium/hydrogen exchanger 2, NHX2	sp|Q56XP4	333	5E-103	381.908	429.401	472.443
Vr40252	NAC domain-containing protein 72, NAC72	sp|Q93VY3	350	2E-114	73.946	21.275	61.914
Vr44893	NAC domain-containing protein, NAC1	sp|Q84TE6	339	5E-112	24.715	17.499	19.606
Vr56348	NAC transcription factor, NAC2	sp|Q8GY42	303	3E-97	7.724	1.993	6.702
Vr34452	NAC transcription factor NAM-B2, NAMB2	sp|A0SPJ6	188	9E-53	105.389	35.786	100.297
Vr32636	Auxin-responsive protein IAA9, IAA9	sp|Q38827	404	8E-132	167.645	180.603	152.081
Vr13386	Auxin-responsive protein IAA26, IAA26	sp|Q8LAL2	227	9E-67	95.846	129.343	104.800
Vr32189	Auxin-responsive protein IAA14, IAA14	sp|Q38832	337	7E-111	630.691	551.345	220.222
Vr13709	Auxin-responsive protein IAA8, IAA8	sp|Q38826	207	1E-58	112.586	79.588	82.916
Vr38087	Alcohol dehydrogenase 1, ADH1	sp|P13603	618	0	7170.197	7091.752	3678.503
Vr13399	Alcohol dehydrogenase, ADH	sp|P13603	307	5E-101	52.076	202.286	151.123
Vr33063	Histidine kinase 1, AHK1	sp|Q9SXL4	847	0	28.731	1.089	5.825
Vr33358	Histidine kinase 2, AHK2	sp|Q9C5U2	1275	0	20.795	12.318	11.801
Vr40153	Histidine kinase 3, AHK3	sp|Q9C5U1	1351	0	49.501	35.556	34.619
Vr38180	Dehydration-responsive protein RD22, RD22	sp|Q08298	310	2E-99	1249.984	356.157	738.560
Vr34639	Auxin-induced protein 22E, AUX22E	sp|O24543	379	8E-129	52.651	50.996	26.642
Vr31234	Auxin-induced protein 22B, AUX22B	sp|P32294	279	3E-88	58.082	58.968	37.863
Vr39198	Auxin-induced protein 22C, AUX22C	sp|O24541	268	5E-89	6.366	43.768	4.778
Vr47043	Auxin-induced protein 15A, AUX15A	sp|P33081	85.9	8E-20	1.261	39.716	2.638
Vr22610	Cationic peroxidase 1, PER1	sp|P22195	324	2E-107	0.193	139.508	372.845
Vr21159	Auxin efflux carrier component 1, PIN1	sp|Q9C6B8	464	2E-156	0.317	15.078	10.054
Vr39799	MYB transcription factor MYB134	sp|Q6R0H1	122	2E-28	249.382	5.232	112.908
Vr40489	MYB transcription factor MYB114	sp|Q6R0H1	118	5E-28	223.864	4.108	94.885
Vr31128	Cationic peroxidase 2 , PER2	sp|P22196	485	1E-168	4.195	169.179	226.783
Vr36822	Quinone oxidoreductase-like protein, QORL	sp|Q9ZUC1	378	1E-124	7.085	40.927	4.776
Vr39707	Peptidyl-prolyl cis-trans isomerase CYP20, CYP20	sp|Q9ASS6	289	9E-94	58.173	62.356	58.411
Vr38164	Eukaryotic translation initiation factor 5A, eIF5A	sp|Q9AXJ4	313	4E-105	606.853	602.711	602.154
Vr31451	Actin-related protein 4, ACTIN	sp|Q84M92	712	0	18.727	19.322	20.725

Primers for q-PCR verification

Vr39707 F: 5' TCCCCAAACAGCCGAAAA 3' 59.5
Vr39707 R: 5' CCCCTTGAATCATGAAATCCTT 3' 59.9 107bp
Vr38164 F: 5' ACCTTAGGCTTCCAACCGAT 3' 58.0
Vr38164 R: 5' CTCCCATTGCAGACATAACAGA 3' 57.8 99bp
Vr31451 F: 5' CGTGTTTCCTTCTGTTGTTGG 3' 58.1
Vr31451 R: 5' CCTCTTTCCCTTAGCCTTGTC 3' 57.7 142bp
Vr37254 F: 5' ATCTCTCATCCGCAACACTCCT 3' 60.3
Vr37254 R: 5' TTCACCCCCACTTCTCTTACCTC 3' 61.4 123bp
Vr35374 F: 5' ACCTTGTCGCTGTCTTCGTGG 3' 62.4
Vr35374 R: 5' GTGGCGGCTGTTAAATTCCTC 3' 61.4 110bp
Vr35831 F: 5' CCGCAGCCAACACAAAGAAAC 3' 63
Vr35831 R: 5' GGTGATGGATAGAGGCCACGTC 3' 63.1 155bp
Vr29470 F: 5' CTTGTTCTAAGTGCCCTTGTGG 3' 59.3
Vr29470 R: 5' GCTTGCGTTTGCGTTTCCT 3' 61.3 175bp
Vr13756 F: 5' AAGGTTGGCTGGGATGAAT 3' 58.7
Vr13756 R: 5' TAAGGGGAAATGGAGATGGAT 3' 57.1 109bp
Vr36957 F: 5' GCTGGCTATTCTGCTGTACCTG 3' 59.5
Vr36957 R: 5' TCCCTTACTATTGGAGTCGGC 3' 58.7 96bp
Vr24483 F: 5' TTGAGGGAAAGTTGGAAGACC 3' 58.3
Vr24483 R: 5' CCTGAACCTAGAGCAAGGGATT 3' 59.5 196bp
Vr23260 F: 5' ACTCCAACGCCTTCTCTCTCCTC 3' 63.1
Vr23260 R: 5' ATCCCAATCACCTTCTCCCACA 3' 62.9 136bp
Vr34322 F: 5' CAATGGATAAGTTGCCCTCAC 3' 57.2
Vr34322 R: 5' ACCGCTGGTTGGAGATGG 3' 58.5 119bp
Vr39013 F: 5' CAAGGCTCAGAGACCCCG 3' 58.4
Vr39013 R: 5' TAGCAGCAGCGTGACATCG 3' 58.8 126bp
Vr40540 F: 5' ATCTCAGGGAACAGGCAGGTC 3' 60.4
Vr40540 R: 5' TCCGTCTGGAACCAACTCTGA 3' 60.2 121bp
Vr38129 F: 5' CTGAACTGCGAACCAATGC 3' 57.1
Vr38129 R: 5' TTCTTGCGTTTAGGAGGTGG 3' 58.0 136bp
Vr40252 F: 5' AGACCCTCTTGCCCAGTTG 3' 57.1
Vr40252 R: 5' CCGAAAATCGCCTTGCTT 3' 58.7 180bp
Vr44893 F: 5' AGGGTTCAGGTTCCATCCAA 3' 59.0
Vr44893 R: 5' GAATATCCCAAGGTTCGCACT 3' 58.5 125bp
Vr56348 F: 5' ATTCAACCTCAAAACCCCCT 3' 57.6
Vr56348 R: 5' CACAACACCCAATCATCAAGC 3' 58.5 76bp
Vr34452 F: 5' CCGACGAGGAATTGGTTGTG 3' 61.0
Vr34452 R: 5' GAGCGGTTGCCGTTAGGATA 3' 60.4 181bp
Vr32636 F: 5' GACTGTAGCGTCTGGTTCTTCG 3' 59.3
Vr32636 R: 5' CCTCAACTCCGTAGCCTTCAAA 3' 61.3 181bp
Vr13386 F: 5' ACCCCACCAAGAGAAAGGAAG 3' 59.8
Vr13386 R: 5' CCCACTAAGCGTGCAAATAAAA 3' 60.2 128bp
Vr32189 F: 5' GTGGAAACACCAAGGGCTACTG 3' 61.2
Vr32189 R: 5' CCAACCAACCACTTGAGCCT 3' 59.7 171bp

Vr13709 F: 5' TGGGCATAGGAGAGGAGGA 3' 57.5
Vr13709 R: 5' GAGAAAGACGGAACGGAGC 3' 57.0 163bp
Vr38087 F: 5' AGAATCCGCTGTTTCCTCGT 3' 58.8
Vr38087 R: 5' ACAGGTTGCTCTCTTCCGATT 3' 58.1 162bp
Vr13399 F: 5' AGGCTGGTGAAGTGCGTTT 3' 57.9
Vr13399 R: 5' TATGCGAGGAAACAATGGAGT 3' 57.4 101bp
Vr33063 F: 5' ACAAGAGCACCACCCCAAAA 3' 60.2
Vr33063 R: 5' TGTGTAAATCTTCGTGAAATGCC 3' 59.7 194bp
Vr33358 F: 5' GCATCAGGGGGGGTGTTTG 3' 62.9
Vr33358 R: 5' GCCTCAGCACGGCTTTTCAG 3' 63.2 125bp
Vr40153 F: 5' CAAATCGTCTGGGGGTTATCC 3' 60.8
Vr40153 R: 5' GAAGCAGTTTCTCCACCAATGAC 3' 60.5 144bp
Vr38180 F: 5' CCCGAGTCAGAGGAGGCTAA 3' 59.3
Vr38180 R: 5' CCCCGTTTCTTTGTCAACTTCT 3' 60.2 177bp
Vr34639 F: 5' AACTCTGATTCTTCGGACTCCACAAC 3' 64.3
Vr34639 R: 5' TCGCCCTGCTCCACCTTTTT 3' 64.3 143bp
Vr31234 F: 5' GCTCCCAGGAACAGAAGAAA 3' 56.8
Vr31234 R: 5' ATAAATCCCAGCACCCTCAT 3' 56.1 199bp
Vr39198 F: 5' TGGCCTCCGGTTTGCTCCTA 3' 65.1
Vr39198 R: 5' CCGCATCCTTCAACGCTTCG 3' 66.1 193bp
Vr47043 F: 5' TCAAGTTTTGGCAAGAAGCA 3' 57.0
Vr47043 R: 5' GTTCTCCCCAACATAGACAGC 3' 56.2 90bp
Vr22610 F: 5' CAAGAATCTCATCAACCAAAAGGG 3' 62.1
Vr22610 R: 5' GGGCTAAGGTTTCCCATTTTG 3' 60.5 156bp
Vr21159 F: 5' ATGCGGAGATTTACTCGCTTCA 3' 61.7
Vr21159 R: 5' CCCCCCCAAAACTACTCTGC 3' 61.0 132bp
Vr39799 F: 5' AAAAGGAAGAGCCCAACAAAG 3 '58.2
Vr39799 R: 5' CTGGAGAATAGCGCCTGAAA 3' 58.2 145bp
Vr40489 F: 5' GGACCTGCTTCATCTGTATCACTG 3' 60.5
Vr40489 R: 5' TGTTTGTGGTCTCCGTCATCTAA 3' 59.8 164bp
Vr31128 F: 5' CCTAGACACGGGTAGTCAAAGAGC 3' 61.6
Vr31128 R: 5' TCATCGTTCCAGAGGGCTTG 3' 60.7 102bp
